# The paradox of closing mutual funds to new investors

**DOI:** 10.1371/journal.pone.0290254

**Published:** 2023-09-14

**Authors:** Svetoslav Covachev

**Affiliations:** Institute of Finance, Corvinus University of Budapest, Budapest, Hungary; University of Southampton - Malaysia Campus, MALAYSIA

## Abstract

I provide an explanation for the well-established paradox that mutual funds that close to new investors fail to maintain their pre-closure positive abnormal performance after closing. Using a large sample of active US equity mutual funds, I find that the within-style performance ranks of closed funds revert to the mean as strongly as those of a control group of open funds. Furthermore, I show that funds that are closed do not drastically alter their investment allocations and hold portfolios that are indistinguishable from their portfolios prior to closing in terms of risk-adjusted performance. Therefore, mean reverting stock returns explain the deterioration in fund performance after closing to new investors. I also document that funds are not capable of better preserving their assets when anticipating a decline in performance by closing to new investors and thus adding exclusivity. Furthermore, the control group of open funds does not suffer from excessive flows. Closing to new investors is at best unnecessary from the stand-alone perspective of the fund.

## 1. Introduction

Mutual fund managers have a clear incentive to maximize the value of the assets under management of their funds. Unlike hedge funds that typically charge a performance fee, mutual funds typically only charge investors a fixed percentage of the funds’ assets. Therefore, fund performance is only important to the commercial success of a mutual fund to the extent that it facilitates asset growth. It is therefore puzzling as to why a mutual fund might decide to stop accepting money to manage from new investors. Such action is likely detrimental to asset growth, at least in the short term, and is likely to result in lower dollar amounts of management fees [1]. Yet, the closing of funds to new investors is not uncommon in the mutual fund industry [2].

At first glance, the main motivation for mutual funds to close to new investors is to preserve good performance in the face of diseconomies of scale. Closing a fund to new investors results in a reduction in the growth of the total amount of money that the fund managers must invest, which may enable them to maintain their preferred investment style by avoiding capacity constraints [1, 2]. The preservation of good performance would enable organic growth of assets under management and may even entice existing investors to devote more money to the fund. Furthermore, consistent good performance enhances the long-term reputation of the manager, the fund and even the fund family, which may translate into economic gains. It is therefore credible that performance preservation is a sincere intention of fund managers who close funds to new investors. What is surprising is that there is almost no empirical evidence in the literature for the closure of funds to be an effective performance conservation tool. This is particularly puzzling given that there is a large body of empirical evidence for diseconomies of scale in the mutual fund industry, for example, see [3]. Furthermore, diseconomies of scale are an integral part of the rational model of the fund management industry of Berk and Green [4].

The objective of this study is to provide an explanation for why mutual funds are unable to sustain their pre-closure performance after closing to new investors. Previous studies have extensively documented this phenomenon [5–9]. However, their focus is on identifying non-performance related motivations for closing mutual funds to new investors [7–9]. This is, to the best of my knowledge, the first study that explores the factors that prevent mutual funds that are closed to new investors from preserving their superior performance.

First, I use a large contemporary sample of active US equity mutual funds to add to the existing evidence that mutual fund performance deteriorates after closure. However, after controlling for size and past performance, I do not find evidence that closed funds underperform their open peers. More specifically, I demonstrate that the within-style performance ranks strongly revert to the mean, regardless of whether the funds are closed or not. This result adds to the body of evidence that closed mutual funds do not underperform relative to their counterfactuals, for example, see [6]. I then proceed by comparing the gross fund performance to the performance of a “ghost” portfolio of the stocks that the fund held prior to closing to new investors (inspired by Lapatto and Puttonen [10]), constructed using the original portfolio weights. I do not find evidence that the risk-adjusted performance of the two differs substantially. Furthermore, most of the actual fund portfolio after the closure is invested in the same stocks as the “ghost” portfolio. This demonstrates that funds do not drastically modify their investment approaches when closing. Therefore, mean reverting stock returns must drive the performance difference between before and after the fund closing event.

I also examine the investment dynamics of the funds around their closures to new investors. Funds that subsequently close hold a higher percentage of cash relative to their same style peers of similar size. Most of the excess cash is invested following the closures, resulting in an increase in both the number of stocks in the funds’ portfolios and the average ownership share of the funds in the stocks of their portfolios. This result is indicative of liquidity constraints increasing and deterring funds from disinvesting in the stocks that discontinue to outperform due to mean reversion.

Finally, I explore a defensive strategy hypothesis, according to which, mutual funds close to signal exclusivity and better preserve their size during the subsequent deterioration in performance. This strategy would be effective if it results in higher new sales to current shareholders and lower redemptions which more than offset the loss of new sales to new investors. I find no evidence that supports this hypothesis. After controlling for fund size and the within-style past performance rank, closed funds exhibit lower overall new sales and higher redemptions. The former holds for all performance quintiles, whereas the latter holds for all but the top performance quintile in which redemptions are invariant to whether the fund is closed or not. Furthermore, I find that the average monthly net cash flow in the matched sample of open funds is only 0.06%, which indicates that the closed funds would not have faced excessive flows had they remained open.

The paper continues as follows. In Section 2, I review previous articles on funds that close to new investors. In Section 3, I formulate the hypotheses. Section 4 contains a description of the data and an analysis of summary statistics. Section 5 presents the main empirical methods used. In Section 6, I analyze the results. Section 7 concludes the article.

## 2. Related literature

Early studies on mutual funds showed that, although mutual funds close to new investors following superior performance, they are incapable of maintaining abnormally high performance after the event [5, 6]. These findings are surprising, because they are in sharp contrast with the typical stated intentions of mutual fund managers when they announce the closures. Fund managers claim that the main motivation for closing their funds to new investors is to preserve their outperformance. Therefore, these early findings produced an unresolved puzzle which was addressed in later studies. Furthermore, the small sample size of these studies is an obvious limitation. In particular, the sample of Smaby and Fizel [5] consists of only 25 closed funds despite the study covering the entire decade from 1982 to 1991. Likewise, Manakyan and Liano [6] analyzed only 27 funds that closed during the 1978 to 1994 period.

Later studies focused on providing rational explanations for mutual funds closing to new investors in light of their failure to continue outperforming subsequently. To the best of my knowledge, only Bris et al. [8] provide a rational explanation at the individual fund level. According to the authors, closing a mutual fund to new investors is a mechanism used by fund managers to extract economic rents in the form of higher gross advisory fees following periods of high performance and large inflows. Bris et al. [8] show that the superior performance is not sustained following the closure of the fund and interpret the increased fees as a substitute for the managerial fees foregone by restricting inflows. Therefore, the authors argue that the fund investors do not benefit from this arrangement at all.

Other studies have focused on the incentives to close a fund to new investors at the fund family level. Zhao [7] documented a fund flow spillover effect arising from the closing of a fund. The author found evidence that mutual fund families that have a recently closed star fund benefit from additional investment inflows across their remaining funds. Hence, Zhao [7] interprets the decision to close a mutual fund as a mechanism to send a signal of outstanding performance which attracts the attention of investors to the entire fund family. Furthermore, this is the first study on closed mutual funds with a reasonably large sample consisting of 139 closed funds. Chen et al. [9] also found evidence that the primary motivation to close funds to new investors is often at the fund family level. The authors show that some fund families close and clone funds in order to attract investors’ flows to the cloned funds and charge higher management fees in the clones relative to the original funds. This behavior is interpreted as rent seeking. Conversely, Chen et al. [9] find that the funds which close without cloning are able to sustain their pre-closure superior performance. Thus, this is the first study that finds evidence that the closing decision can be good for the fund shareholders, which is in line with the statements made by fund managers. Overall, these studies shed light on contrasting motivations for closing mutual funds to new investors.

## 3. Hypotheses formation

### 3.1. Mean reversion at the fund level

Mean reverting mutual fund performance is a well-established broad phenomenon [11, 12]. I hypothesize that mean reversion is driving the performance deterioration following the closure of equity mutual funds to new investors. Previous studies concur that fund performance is superior prior to closing relative to peers that do not close. Superior performance is normally attributable to either luck or skill. In the case of luck, mean reversion is inevitable in the long-run. Even in the presence of superior skill, mean reversion of performance can occur if the skill level itself is mean reverting. This would be the case if the value of some investment skills depreciates over time as other market participants gradually catch up, since obtaining abnormal returns is a zero-sum game. There is evidence in the literature that some market anomalies that can be exploited to obtain abnormal returns indeed diminish over time. For example, Jones and Pomorski [13] show that the well-known January effect and the short-term autocorrelation of the returns of market indices have gradually diminished.

I do not discriminate between the luck and diminishing skill explanations for mean reversion in the performance of closed funds. However, both explanations are inconsistent with the stated purpose of closing a fund to sustain superior performance in the face of diseconomies of scale. The decision to close cannot conceivable alleviate either of these drivers of performance mean reversion. However, the managers that close their funds to new investors may not be fully aware of mean reversion. Tversky and Kahneman [14] argue that while regression towards the mean is very common, it is often unnoticed or misunderstood. The authors claim that this is due to the representativeness bias, according to which extreme observations lead to extreme future predictions. It is therefore conceivable that fund managers make sincere but overly optimistic predictions about future performance when they decide to close the fund after achieving abnormal returns for an extended period of time. Mutual funds investors also seem to extrapolate future fund performance from historic returns, according to the flow-performance relation, which is particularly strong for top performing funds, according to Sirri and Tufano [15].

### 3.2. Mean reversion at the stock level

De Bondt and Thaler [16] show that stocks with abnormally high past performance (“winners”) subsequently underperform stocks with abnormally low past performance (“losers”) over horizons of up to 5 years. This is in contrast to horizons of 3 to 12 months, during which the “winners” tend to outperform, as in [17]. I further hypothesize that the mean reversion in the performance of funds after they close to new investors is driven by mean reverting stock returns. An equity mutual fund with exceptionally high past performance, such as the typical fund that is about to close to new investors, will hold a portfolio of “winners” going forward, unless it drastically reallocates its capital. Funds that close are typically large, and such reallocations may be too costly to implement, particularly given that liquidity constraints are cited by managers as motivation for the closing decision. Furthermore, the portfolio weights of stocks that outperform increase unless the fund rebalances its portfolio regularly which is also costly. Therefore, I expect that many funds continue to hold investment portfolios that are heavily tilted towards their past “winners” after they close, which would explain why they subsequently fail to outperform.

### 3.3. Defensive strategy

I also examine a defensive strategy hypothesis as a potential explanation of why mutual funds close to new investors in the absence of evidence of superior performance after the event. Let us suppose that some mutual fund managers expect performance to deteriorate going forward after a period of very strong performance. Assuming a convex flow-performance relationship, as in [15], these fund managers should not expect strong inflows after investors observe the deterioration in performance. Therefore, closing the funds to new investors might not be too costly in terms of foregone new sales. It is possible that closing the funds boosts new sales to current investors and reduces their redemptions from the funds. If these two potential effects more than offset the loss of new sales, then mutual fund managers would have a strong incentive to close their funds to new investors.

The defensive strategy hypothesis raises the question of how can closing a fund to new investors boost net flows. This could occur if investors value exclusivity. The idea is comparable to a social club that is very sought after because there are very strict restrictions on who can join. In the context of mutual funds, taking money out of a fund may already be stressful due to fear of missing out on subsequent performance. This may be why investors do not as readily disinvest from poorly performing funds as they invest in the top performing funds, as in [15]. The fear of missing out is likely increased by the irreversibility of the decision to take their money out of a fund that is closed to new investors. Investors do not typically have the chance to change their minds later and reinvest in the fund. Furthermore, a shareholder of a closed fund can give shares of the fund to non-shareholders, thus making them shareholders and giving them the chance to buy more shares [18]. Through this channel, a closed fund can have new investors, thus increasing its new sales. In addition, this potentially valuable option and the fear of missing out can impede current investors from disinvesting from the fund and thus dampen its redemptions.

Closing a fund to new investors can also augment net flows through signaling. The decision to close could be a costly, and hence credible, signal of superior fund governance as argued by Chen et al. [9]. While investors who receive this signal may consider investing in other funds that are managed by the same manager or fund family, they would likely prefer to invest in the closed fund itself if possible. As previously mentioned, both shareholders and non-shareholders of a closed fund can effectively invest in the fund. Therefore, the negative impact of the additional barrier to invest in the fund may be outweighed by the positive effect of the signal on fund flows.

## 4. Data and summary statistics

The primary data source is The Center for Research in Security Prices (CRSP) Survivorship Bias Free Mutual Fund Database. Data is collected at the share class (“CRSP FUNDNO”) level and aggregated at the fund level using “CRSP PORTNO” as the unique fund identifier. Fund size is the total of the “Total Net Assets” of the share classes belonging to the fund. Fund age is the difference in years between a date and the minimum of the “Fund First Offer Date” values of the fund’s share classes. The remaining quantitative characteristics of a fund are just weighted averages of the quantitative characteristics of its share classes where the weights are the share class sizes. The qualitative characteristics of a fund are the qualitative characteristics of the fund’s largest share class. The only exception is the “Open to Investment Flag” variable of a fund, which is set to “N” only if it is equal to “N” for all share classes of the funds (as in [9]). The sample consists of US equity mutual funds (funds that have a four-digit “CRSP Style Code” that begins with “ED”) from July 2003 to December 2017. Funds that are either explicitly flagged as index funds (“Index Fund Flag” is equal to “B”, “D” or “E”) or contain the word “Index” in the “Fund Name” are removed from the sample. Furthermore, funds that manage less than $10 million or are younger than one year are excluded from the sample, which only affects the subsample of open funds, since these funds are not closed to new investors. After applying these filters, the remaining sample contains 9,575 unique US equity mutual funds, of which 702 closed to new investors at least once during the sample period. The quantitative fund variables are winsorized at the 0.5% and 99.5% levels in each month of the sample period. The fund data is of monthly frequency, except for “Expense Ratio” and “Turnover Ratio”, which are of annual frequency. The fund holdings data is of either quarterly or monthly frequency, depending on the fund and the time period.

The CRSP Mutual Fund data is supplemented with data from Morningstar Direct. The databases are merged at the share class level by setting “Fund CUSIP” from CRSP equal to the third to eleventh characters of “ISIN” from Morningstar Direct. In the cases where this does not work, the databases are merged using the “Ticker” field, which is available in both databases. To obtain links of “CRSP PORTNO” to “FundId”, the largest share class of a given “CRSP PORTNO” is used. For each fund, the oldest available link is used throughout the entire sample to avoid linking the same CRSP fund to different Morningstar funds on different dates. Morningstar Direct is used to retrieve the “New Sales”, “Redemptions” and “Morningstar Category” time series.

Stock return data adjusted for dividends and splits was downloaded from the CRSP database. I obtained Carhart’s [11] four factors from [19], the five factors of Fama and French [20] from [21] and the q-factors of Hou et al. [22] by sending a request to Lu Zhang.

The summary statistics in Table 1 provide some preliminary insights. Closed (Panel A) and open (Panel B) funds have similar raw performance both before and after fund fees. Open funds have a slightly higher mean monthly net return of 0.79% relative to the 0.71% of closed funds. Not surprisingly, closed funds have a much larger mean size ($2,955 million in TNA versus $1,074 million). Both types of funds have average expense ratios of just above 1.1% per year. Open funds have a slightly higher mean turnover ratio of 0.86 versus 0.73, whereas closed funds have a higher mean age of 16.43 years versus 13.83. Both types of funds tend to belong to relatively large mutual fund families in terms of number of funds and assets under management of the family. It is not surprising that the mean fractional net flow (as defined by Sirri and Tufano [15]) of closed funds is -1.09%, whereas open funds have a mean fractional net flow of 0.32%. Even at the 90^th^ percentile, closed funds have a relatively small positive fractional net flow of 1.18%, which must be due to additional money invested by existing investors, as described in [18]. Panel C contains the differences between the means presented in Panels A and B and the results of the Folded F-test of equality of variances, the Student’s t-test of equality of means that assumes equal variances and the Welch’s t-test that should be used when the equal variances assumption does not hold. The results of the Folded F-test do not support equal variances between the two populations of mutual funds, except in the case of the family total size variable. Nonetheless, all differences in means are statistically significant regardless of which t-test is used.

**Table 1:** Summary statistics. I provide summary statistics for closed funds in Panel A and open funds in Panel B from July 2003 to December 2017. Panel C contains the differences in means between the two groups of funds, the Folded F-test statistics of equality of variances, Student’s t-test statistics and Welch’s t-test statistics. *, **, and *** indicate significance at the 10%, 5%, and 1% levels, respectively. All variables are taken directly from the CRSP Mutual Fund database unless otherwise stated. Gross Monthly Return represents the return of a fund before fees and is calculated by adding the annual Net Expense Ratio of the fund, divided by 12, to the Net Monthly Return of the fund. The Net Monthly Return is the return of a fund to its shareholders after fees. Total Net Assets represents the size of a fund and is calculated as the sum of the Total Net Assets of all share classes belonging to the fund. Net Expense Ratio is the proportion of the total investments of a fund that went towards covering its operating expenses and may include waivers and reimbursements. Fund Turnover Ratio is taken directly from the CRSP Mutual Fund database and is defined as the minimum of aggregated sales and aggregated purchases of securities of a fund, divided by the fund’s 12-month average size. Age of Fund is the number of years that have passed since the inception date (“Fund First Offer Date”) of a fund’s oldest share class. Number of Funds in the Family is the total number of funds offered by the management company that oversees a fund. Family Total Size is the sum of the Total Net Assets of all funds belonging to the management company of a fund. Fractional Flow is the Sirri and Tufano [15] measure and is calculated as the monthly growth rate of the Total Net Assets of a fund minus the fund’s Net Monthly Return.

Panel A: Closed funds.							
Variable	Mean	Median	Std Dev	1^st^ Pctl	10^th^ Pctl	90^th^ Pctl	99^th^ Pctl
Gross Monthly Return (%)	0.80	1.23	4.93	-13.13	-5.42	6.10	12.40
Net Monthly Return (%)	0.71	1.14	4.94	-13.25	-5.52	6.00	12.33
Total Net Assets ($ million)	2,955	824	5,614	15	66	8,049	31,029
Net Expense Ratio (% per year)	1.12	1.11	0.38	0.29	0.69	1.54	2.51
Fund Turnover Ratio (annual)	0.73	0.54	0.92	0.02	0.13	1.34	4.29
Age of Fund (years)	16.43	14.27	10.40	2.69	6.25	29.58	51.66
Number of Funds in the Family	61.63	38.00	70.15	1.00	4.00	139.00	410.00
Family Total Size ($ million)	304,307	33,964	926,009	215	1,552	413,438	4,706,701
Fractional Flow (% of TNA)	-1.09	-0.79	4.81	-16.79	-3.71	1.18	7.74
Panel B: Open funds.							
Variable	Mean	Median	Std Dev	1^st^ Pctl	10^th^ Pctl	90^th^ Pctl	99^th^ Pctl
Gross Monthly Return (%)	0.87	1.16	4.49	-12.11	-4.51	5.80	11.74
Net Monthly Return (%)	0.79	1.09	4.49	-12.21	-4.60	5.71	11.65
Total Net Assets ($ million)	1,074	230	2,834	11	26	2,344	15,795
Net Expense Ratio (% per year)	1.15	1.14	0.46	0.08	0.60	1.70	2.47
Fund Turnover Ratio (annual)	0.86	0.58	1.10	0.03	0.15	1.68	6.03
Age of Fund (years)	13.83	11.16	11.99	1.25	2.99	25.91	68.33
Number of Funds in the Family	69.23	43.00	87.42	1.00	3.00	139.00	430.00
Family Total Size ($ million)	290,473	28,352	918,950	21	358	295,313	4,294,542
Fractional Flow (% of TNA)	0.32	-0.40	11.26	-15.63	-2.97	3.80	24.88
Panel C: Closed funds−Open funds.							
Variable				Δ Mean	Folded F	Student t	Welch t
Gross Monthly Return (%)				-0.07	1.21***	-2.15**	-1.96**
Net Monthly Return (%)				-0.08	1.21***	-2.39**	-2.19**
Total Net Assets ($ million)				1881	3.92***	79.38***	42.63***
Net Expense Ratio (% per year)				-0.03	1.43***	-6.64***	-7.82***
Fund Turnover Ratio (annual)				-0.13	1.43***	-13.83***	-16.30***
Age of Fund (years)				2.60	1.33***	27.33***	31.20***
Number of Funds in the Family				-7.60	1.55***	-10.99***	-13.48***
Family Total Size ($ million)				13834	1.02	1.89*	1.88*
Fractional Flow (% of TNA)				-1.41	5.49***	-15.83***	-33.99***

**Fig 1:**
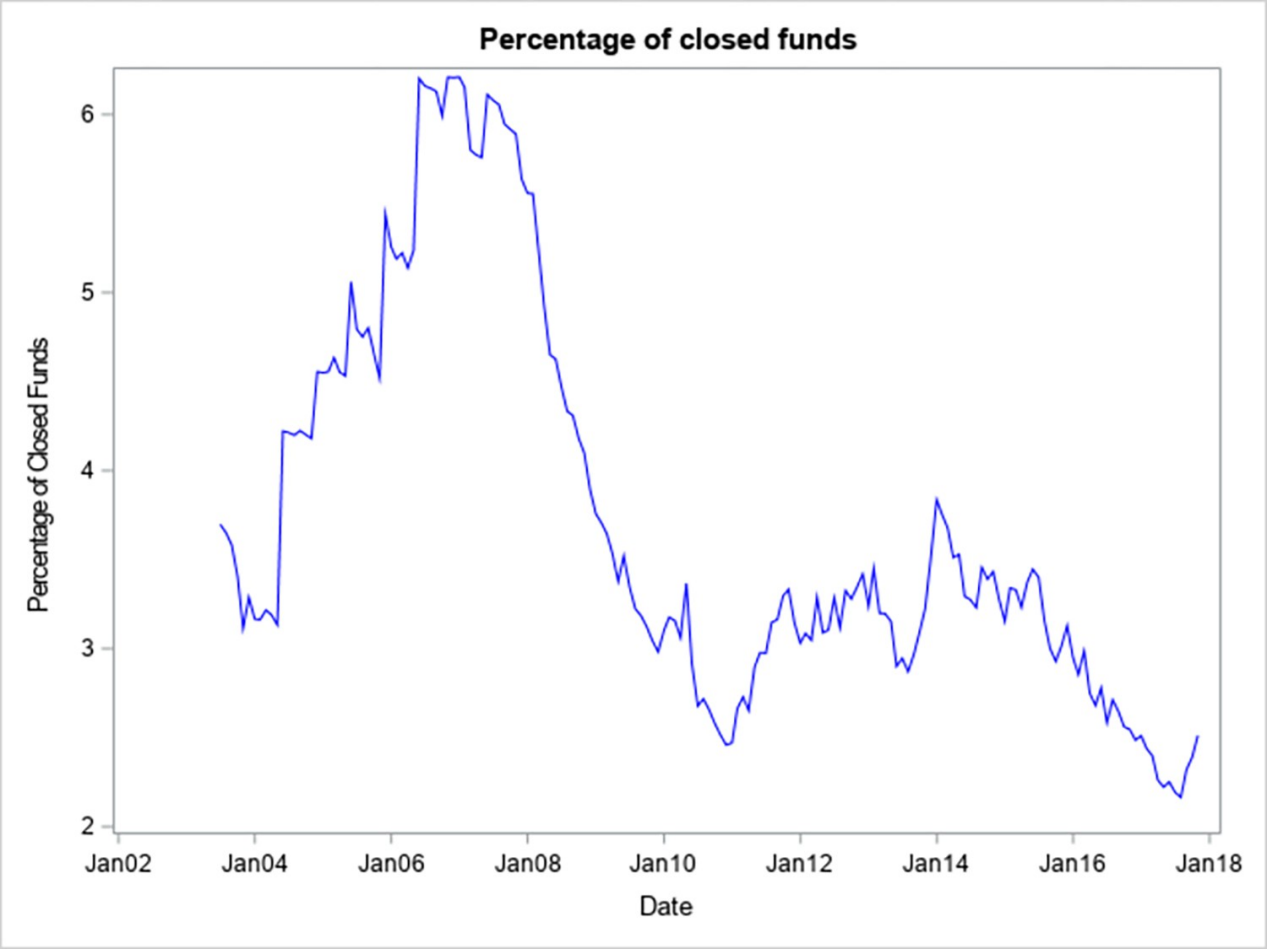
Percentage of closed funds.

**Fig 2:**
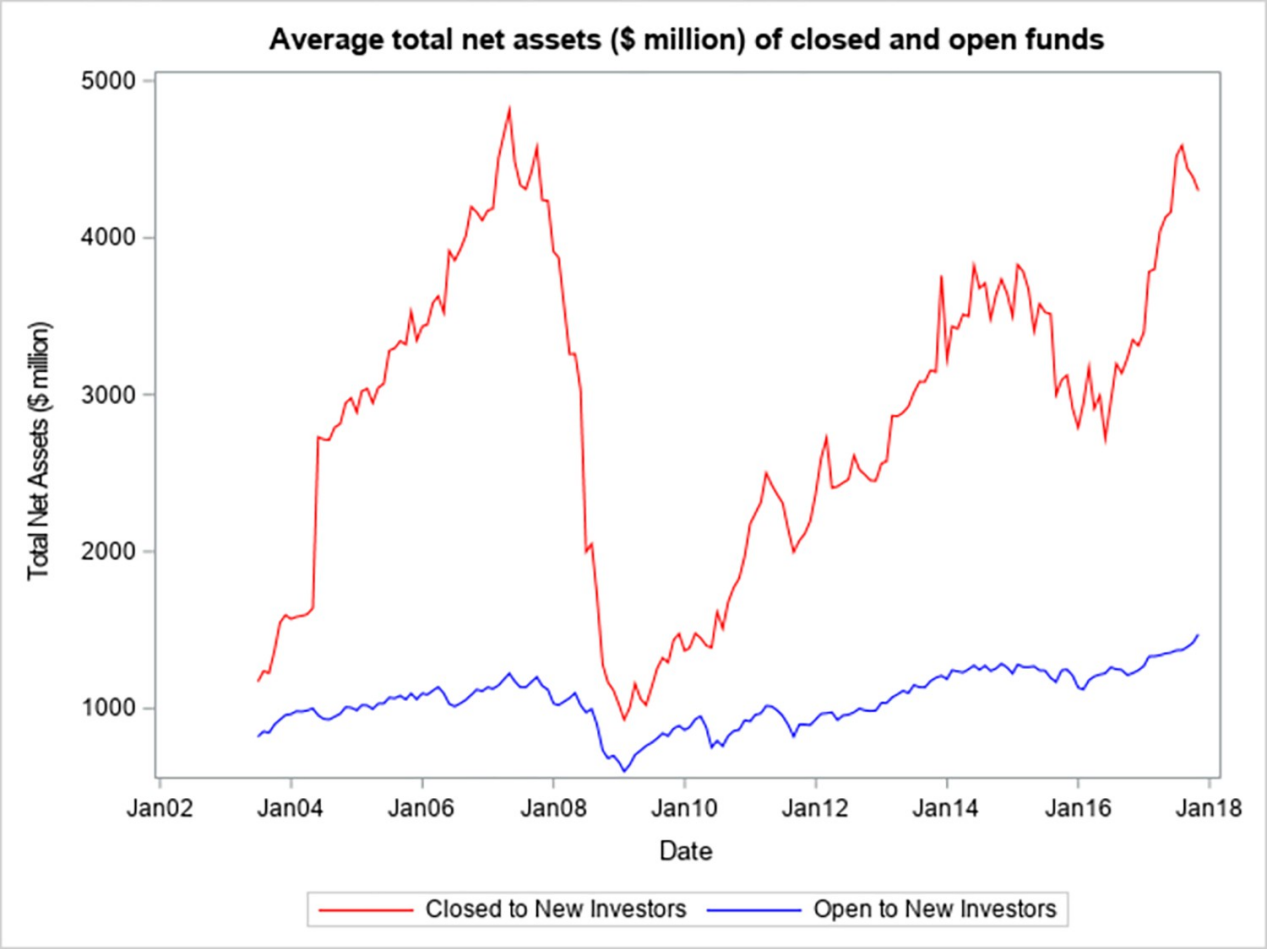
Average total net assets ($ million) of closed and open funds.

Fig 1 shows that the percentage of funds that are closed to new investors appears to be procyclical and has ranged from just over 2% to just over 6%. The peak occurred a few months before the start of the global financial crisis of 2007–2008. Since the end of the crisis, the percentage has been relatively stable. However, the percentage of closed funds has had a slow but steady decline after January 2014. This may be due to tightening competition for flows in the active equity mutual fund segment and (or) the dissemination of academic studies that do not find benefits to investors resulting from the closing of a fund. Fig 2 shows that the average size of funds that are closed appears to be even more procyclical, whereas the average size of open equity mutual funds is relatively stable across different economic conditions.

Fig 1 presents the percentage of active equity mutual funds that have all of their share classes closed to new investors in each month from July 2003 to December 2017. The data source is the CRSP Mutual Fund database.

Fig 2 plots the average total net assets (in $ million) of all sample funds that are closed to new investors and the average total net assets (in $ million) of all sample funds that are open to new investors. The averages are presented for each month from July 2003 to December 2017, and the data is obtained from the CRSP Mutual Fund database.

Table 2, Panel A, shows summary statistics of the number of consecutive months that a mutual fund was open (closed) before (after) closing to new investors. The median fund in the sample was open for at least 24 consecutive months prior to closing. However, the median fund that closed stayed closed for just 8 consecutive months. Panel B shows summary statistics of the number of consecutive months in which a fund remained closed and subsequently remained open, conditional on the fund reopening before the end of the sample period. The median number of months before reopening is 20 (as in [9]), whereas the median number of months without a fund closing again after reopening is 15.

**Table 2:** Summary statistics of consecutive months in which a fund was open (closed). Panel A contains summary statistics of the number of consecutive months in which a fund remained open prior to closing and the number of consecutive months in which the fund subsequently remained closed. Panel B contains summary statistics of the number of consecutive months in which a fund remained closed prior to reopening and the number of consecutive months in which the fund was subsequently open.

Panel A: Conditional on fund closing.									
	Mean	Median	Std Dev	1^st^ Pctl	10^th^ Pctl	25^th^ Pctl	75^th^ Pctl	90^th^ Pctl	99^th^ Pctl
Open	28	24	23	1	1	8	42	62	88
Closed	15	8	16	1	1	2	26	39	71
Panel B: Conditional on fund reopening.									
	Mean	Median	Std Dev	1^st^ Pctl	10^th^ Pctl	25^th^ Pctl	75^th^ Pctl	90^th^ Pctl	99^th^ Pctl
Closed	24	20	19	1	3	9	37	56	77
Open	21	15	22	1	1	1	30	54	85

Table 3 contains a category breakdown of the closed and open equity mutual funds in the sample. More than half (57.29%) of the funds that are closed are small cap funds, whereas less than a quarter (22.80%) of open funds belong to this segment. Furthermore, nearly half (49.08%) of the closed funds belong to the “Growth” style category, compared to just 42.79% of open funds. The intersection of these two categories, “Small Growth”, contains 29.50% (9.90%) of all closed (open) funds. Since small cap growth funds are likely to be the most liquidity constrained (as in [3]), this breakdown is consistent with the diseconomies of scale argument for closing a fund to new investors.

**Table 3:** Category breakdown of closed and open funds. I compare the category breakdown of funds that are closed to new investors to that of funds that are open to new investors. Each figure represents the percentage of funds which are closed (open) that belong to a given category. The categories are obtained from the “Morningstar Category” variable from the Morningstar Direct database. Panel A contains the breakdown by size categories which indicate the size of the market capitalization (market cap) of the stocks that a fund typically invests in. Panel B shows the breakdown by style categories that are indicative of the type of stocks that a fund invests in. Panel C reports the breakdown by the categories which are intersections of the size and style categories. The t-stats of the average differences are reported in parentheses.

Panel A: Size Category (%).				
	Small	Mid-Cap	Large	Total
Closed	57.29	20.84	21.87	100
Open	22.80	21.80	55.40	100
Difference	34.49	-0.96	-33.53	0
	(66.44)	(-2.32)	(-100.33)	
Panel B: Style Category (%).				
	Growth	Blend	Value	Total
Closed	49.08	28.44	22.48	100
Open	42.79	31.66	25.55	100
Difference	6.29	-3.22	-3.07	0
	(15.13)	(-13.08)	(-7.47)	

Panel C: Size-Style Category (%).										
	Small Growth	Mid Growth	Large Growth	Small Blend	Mid Blend	Large Blend	Small Value	Mid Value	Large Value	Total
Closed	29.50	9.22	10.36	14.94	5.47	8.03	12.85	6.15	3.49	100
Open	9.90	11.36	21.52	7.96	5.60	18.10	4.94	4.84	15.77	100
Difference	19.60	-2.14	-11.16	6.98	-0.13	-10.07	7.91	1.31	-12.28	0
	(96.80)	(-7.25)	(-39.62)	(39.17)	(-1.20)	(-67.17)	(23.52)	(6.52)	(-71.98)	

## 5. Methodology

### 5.1. Fund performance after closure relative to before

I start by testing whether fund performance deteriorates after the fund closes to new investors as according to previous studies. I use pooled OLS regressions in which the dependent variable is fund performance, and the independent variable is a dummy variable equal to one if the fund is closed to new investors and zero otherwise. I exclude from this regression funds that never close as well as all data after funds reopen to new investors, even if they subsequently close again. The fund performance measures are excess net return and the four-factor, five-factor and q-factor model alphas. The mean measures for each date and fund category combination are deducted from the corresponding fund measures. The control variables are fund characteristics including the Pollet and Wilson [23] average ownership share and the Sirri and Tufano [15] fractional flow at the quarterly level. All controls are lagged by one month, except for the annual variables which are lagged by one year. The general form of the regressions is:

$${Perf}_{i,t}=\alpha +\beta_{1}{Closed}_{i,t}+\sum_{j=2}^{J}\beta_{j}X_{j,i,t}+\varepsilon_{i,t},$$ (1)

where Perf_i,t_ is the style-adjusted performance of fund i in month t, α is a constant, β_1_ is a slope coefficient that measures the marginal effect of closing a fund to new investors on the style-adjusted performance of the fund, Closed_i,t_ is a dummy variable equal to one if fund i is closed in month t and zero otherwise, β_j_ is the marginal effect of the j^th^ explanatory variable, X_j,i,t_ is the value of the j^th^ explanatory variable of fund i in month t and ε_i,t_ is the idiosyncratic error term of fund i in month t.

I obtain fund alphas using the Carhart [11] methodology. More specifically, I regress the monthly excess net returns of a fund in months t-1 to t-12 on the contemporaneous monthly factors to obtain the factor loadings of the fund in month t. The alpha of the fund in month t is equal to the difference between the excess net return of the fund and the sum of the products of the factor loadings and the respective factor returns in month t. I also compute the factor loadings with daily data instead of monthly data as a robustness check, and the results are very similar.

### 5.2. Fund performance after closure relative to peers

I proceed by comparing the performance of funds that are closed to new investors to that of funds with similar past performance and size that are open. For every month, year and “Morningstar Category” (style) combination, all funds are split into five groups using the lagged (by one month) 12-month cumulative net return quintiles. For each month and year combination, all funds are split into five groups based on the size quintiles. The funds are then placed into 5x5 groups based on the intersections of the groups described above. The funds are also split into five performance ranks based on their contemporaneous monthly net return quintiles within each month, year and style category combination. The average contemporaneous within-style performance ranks of closed funds are compared to those of open funds that belong to the same group based on within-style past performance and size.

I also compare the short-term performance persistence of closed funds to that of open funds in a regression framework. I use pooled OLS regressions in which the dependent and control variables are identical to those described in Section 5.1. However, I include in these regressions all fund data remaining after applying the filters described in Section 4. Furthermore, in addition to the closed dummy variable, I include past performance variables as independent variables. These variables are 12-month geometric average excess net return and 12-month geometric average four-factor, five-factor and q-factor model alphas, all of which are lagged by one month and style category demeaned. I also interact each past performance variable with the closed dummy variable. The slope coefficients of the past performance variables are estimates of the performance persistence of open funds, whereas the slope coefficients of the interaction variables are estimates of the performance persistence of closed funds that is incremental to that of open funds. The general form of the regressions is:

$${Perf}_{i,t}=\alpha +\beta_{1}{Closed}_{i,t}+\beta_{2}{Lag\_perf}_{i,t}+\beta_{3}{Closed}_{i,t}\times {Lag\_perf}_{i,t}+\sum_{j=4}^{J}\beta_{j}X_{j,i,t}+\varepsilon_{i,t},$$ (2)

where Perf_i,t_ is the style-adjusted performance of fund i in month t, α is a constant, β_1_ is a slope coefficient that measures the marginal effect of closing a fund to new investors on the style-adjusted performance of the fund, Closed_i,t_ is a dummy variable equal to one if fund i is closed in month t and zero otherwise, β_2_ is the marginal effect of the style-adjusted past performance of a fund on its style-adjusted contemporaneous performance, Lag_perf_i,t_ is the style-adjusted past performance of fund i in month t, β_3_ is the marginal effect of closing a fund to new investors on the sensitivity of the performance of the fund to its past performance, β_j_ is the marginal effect of the j^th^ explanatory variable, X_j,i,t_ is the value of the j^th^ explanatory variable of fund i in month t, and ε_i,t_ is the idiosyncratic error term of fund i in month t.

### 5.3. Pre-closure fund portfolio performance after closure

I follow by forming a “ghost” portfolio of the stocks that a fund held just before closing to new investors, using the actual portfolio weights, and comparing its performance to the actual performance of the fund before fees following the closure. A similar methodology is used by Lapatto and Puttonen [10] who compare the post-merger performance of an acquirer fund with that of the pre-merger portfolio of the target fund held passively following the merger. The portfolio is formed using the holdings and weights of a fund as of the last report date of the fund prior to the month of closure, provided that it is not earlier than a quarter before the closure. Cases in which the fund is not open to investors in each of the 24 consecutive months prior to closure are excluded. The portfolio is then held passively for the first 24 months following the fund closure without rebalancing as well as with quarterly rebalancing of its weights. Gross fund returns are used for the comparison and must be available for at least the first 12 months after the fund closes to new investors. The performance measures are excess return, four-factor alpha, five-factor alpha and q-factor alpha.

### 5.4. Fund flows after closure relative to peers

Finally, I compare the flows of closed funds to those of open funds, while controlling for size, past performance and investment style (“Morningstar Category”). More specifically, all funds are split into five groups at each month-end based on their size quintiles. The funds belonging to a given style are also allocated to five groups at each month-end based on their cumulative net returns over the 12 months ending in the previous month. This double partitioning results in 25 subgroups of funds. The average monthly flows of closed funds are compared to the average monthly flows of the open funds that belong to the same subgroup. The flow measures are New Sales (%), Redemptions (%) and their difference–Net Cash Flow (%). All measures are simply the respective dollar amounts, as obtained from Morningstar Direct, divided by the size of the fund in the previous month, as in [15], and multiplied by 100.

## 6. Results

### 6.1. Fund performance deterioration

**Fig 3:**
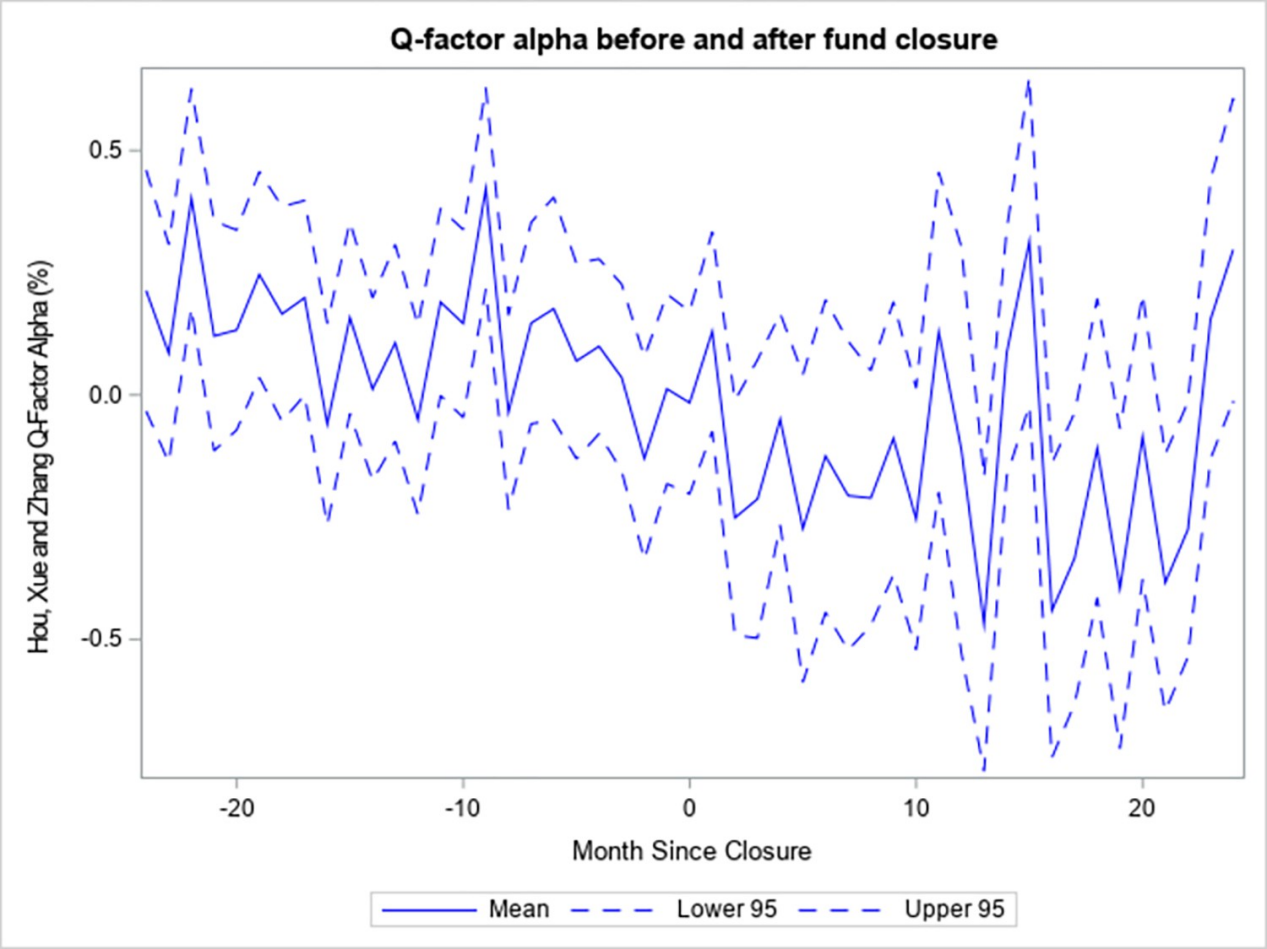
Q-factor alpha before and after fund closure.

The evidence is consistent with the performance of mutual funds that close to new investors deteriorating after the event as according to previous studies. Fig 3 shows the q-factor model alpha estimate of those funds from 24 months before the event to 24 months after. The figure also contains the lower and upper bounds of the 95% confidence intervals. The alpha estimate is positive in most months prior to the closure and negative in most months after. Furthermore, the non-overlapping confidence intervals are indicative of a deterioration in the risk-adjusted performance of mutual funds after they close to new investors.

Fig 3 displays the Hou et al. [22] q-factor model alpha estimates (in percent per month) of funds that close to new investors from 24 months before the closure to 24 months after. The alpha estimates are obtained by regressing the excess net returns of the funds, as of a certain number of months relative to the event, on the contemporaneous monthly factors. Mean is the regression estimate of alpha, Lower 95 is the lower bound of the 95% confidence interval for alpha and Upper 95 is the upper bound. The net returns of the funds are taken as reported by the CRSP Mutual Fund database, and the factors are obtained from Lu Zhang.

The regression approach enables quantifying the marginal impact of closing a fund to new investors. Table 4 displays the results of the pooled OLS regressions of fund performance on the closed dummy variable described in Section 5.1. All standard errors are clustered at the fund level, but control variables and monthly fixed effects are added only to the regressions of columns (5)-(8). Panel A contains the results of the regressions in which all data is included, up to the points at which the funds reopen to new investors. The slope estimate of the closed dummy is negative and statistically significant at the 5% level in all regressions. The estimate of the marginal impact of closing a fund to new investors on the abnormal return of the fund is between 6.4 and 7.8 basis points per month according to the regressions without controls in columns (1)-(4) and between 8.0 and 11.0 basis points according to the regressions with controls. The estimated magnitude of the effect is even larger for small cap growth funds (see S1 Appendix). Furthermore, none of the control variables have a statistically significant impact on fund performance across all models. Panel B shows the results when only fund data that lies within 24 months of the closure events is considered. These results are very similar to those in Panel A. However, the magnitude of the estimated effect size in Panel B is slightly higher regardless of which model is used. This is indicative of the estimated effect on fund performance truly arising from the closing event. Panel C contains the results when funds that lack 12 consecutive months of return data on either side of their closing events are excluded to remove merger or liquidation motivated fund closures from the analysis (akin to [9]). This refinement roughly doubles or triples the magnitude of the estimated impact of closing a fund to new investors on its performance depending on which model specification is used. It appears that funds that are about to merge or liquidate mute the estimated impact of the closing event. They likely exhibit poor performance both before and after closing to new investors which motivates their subsequent mergers or liquidations.

**Table 4:** The marginal effect of closing to new investors on risk-adjusted performance. The table contains the output of pooled ordinary least squares (OLS) regressions with monthly observations. Only funds that close to new investors are included in the estimations. The dependent variable is fund performance. Return is the excess return after fund fees, Alpha-4 refers to the Carhart [11] four-factor model alpha, Alpha-5 refers to the Fama and French [20] five-factor model alpha, and Alpha-Q refers to the Hou et al. [22] q-factor model alpha. Fund alphas are estimated using the Carhart [11] methodology with the funds’ factors loading for each month estimated over trailing 12-month windows. The mean performance measures of each fund category in each month are subtracted from the measures of the corresponding funds. The demeaned measures represent abnormal percentage returns per month. Closed_dummy is a variable equal to one if the fund is closed to new investors and equal to zero otherwise. All other independent variables are lagged by one month, except for the variables Expense_ratio and Turnover, which are lagged by one year. The Av_ownership variable is calculated as in [23] and represents the average ownership share (%) of a fund in the stocks of its investment portfolio. The Fractional_flow_q variable is the Sirri and Tufano [15] fractional flow (%) measure computed at the quarterly level. The observations in Panel A represent the entire within sample period histories of funds that close to new investors up to the points at which they reopen. The observations in Panel B are further restricted to be within 24 months of the closing events. I additionally restrict the sample in Panel C to funds that have return data for each of the 12 months immediately before and after their closings (similar to [9]). Thus, the funds that are most likely to have closed to new investors in anticipation of a liquation or merger are excluded from the sample. The p-values are reported in parentheses. *, **, and *** indicate significance at the 10%, 5%, and 1% levels, respectively.

Panel A: Pooled OLS regressions with all observations prior to the funds reopening.								
	(1)	(2)	(3)	(4)	(5)	(6)	(7)	(8)
	Return	Alpha-4	Alpha-5	Alpha-Q	Return	Alpha-4	Alpha-5	Alpha-Q
Closed_dummy	-0.066*** (0.00)	-0.064** (0.02)	-0.078** (0.01)	-0.068*** (0.01)	-0.080*** (0.00)	-0.110*** (0.00)	-0.092** (0.01)	-0.084*** (0.01)
Av_ownership					0.008 (0.48)	0.002 (0.92)	0.031* (0.06)	0.014 (0.32)
Fractional_flow_q					0.001 (0.16)	0.001 (0.18)	0.002* (0.05)	0.001* (0.07)
Log_TNA					0.014 (0.10)	0.031*** (0.00)	0.010 (0.40)	0.012 (0.21)
Log_famsize					0.006 (0.11)	0.001 (0.80)	-0.001 (0.91)	-0.002 (0.62)
Age					0.000 (0.93)	-0.001 (0.63)	0.002* (0.07)	0.000 (0.71)
Expense_ratio					-0.093** (0.02)	-0.109** (0.03)	-0.048 (0.36)	-0.064 (0.18)
Turnover					0.021 (0.41)	-0.031 (0.35)	0.005 (0.88)	-0.022 (0.46)
Constant	0.067*** (0.00)	0.068*** (0.00)	0.085*** (0.00)	0.075*** (0.00)	-0.064 (0.46)	-0.021 (0.85)	-0.020 (0.85)	0.030 (0.76)
Clustered S.E.	Fund	Fund	Fund	Fund	Fund	Fund	Fund	Fund
Monthly F.E.	No	No	No	No	Yes	Yes	Yes	Yes
Observations	23084	17994	17994	17994	15605	15558	15558	15558
Panel B: Pooled OLS regressions with observations that are within 24 months of the closings.								
	(1)	(2)	(3)	(4)	(5)	(6)	(7)	(8)
	Return	Alpha-4	Alpha-5	Alpha-Q	Return	Alpha-4	Alpha-5	Alpha-Q
Closed_dummy	-0.087*** (0.00)	-0.070** (0.03)	-0.100** (0.01)	-0.100*** (0.00)	-0.094*** (0.00)	-0.115*** (0.00)	-0.116** (0.01)	-0.115*** (0.00)
Av_ownership					0.003 (0.86)	0.017 (0.41)	0.045* (0.05)	0.025 (0.14)
Fractional_flow_q					0.001 (0.15)	0.001 (0.21)	0.002* (0.06)	0.002* (0.06)
Log_TNA					0.029** (0.01)	0.038*** (0.01)	0.009 (0.57)	0.014 (0.24)
Log_famsize					0.001 (0.90)	-0.001 (0.88)	0.002 (0.74)	-0.001 (0.87)
Age					-0.001 (0.63)	-0.001 (0.44)	0.001 (0.44)	-0.001 (0.71)
Expense_ratio					-0.067 (0.16)	-0.060 (0.31)	0.019 (0.81)	-0.014 (0.80)
Turnover					0.017 (0.56)	-0.015 (0.70)	-0.036 (0.44)	-0.024 (0.54)
Constant	0.076*** (0.00)	0.072*** (0.00)	0.100*** (0.00)	0.090*** (0.00)	-0.132 (0.22)	-0.099 (0.42)	-0.056 (0.70)	-0.058 (0.59)
Clustered S.E.	Fund	Fund	Fund	Fund	Fund	Fund	Fund	Fund
Monthly F.E.	No	No	No	No	Yes	Yes	Yes	Yes
Observations	14137	11204	11204	11204	9717	9674	9674	9674
Panel C: Pooled OLS regressions with observations that are within 24 months of the closings (reduced sample).								
	(1)	(2)	(3)	(4)	(5)	(6)	(7)	(8)
	Return	Alpha-4	Alpha-5	Alpha-Q	Return	Alpha-4	Alpha-5	Alpha-Q
Closed_dummy	-0.283*** (0.00)	-0.220*** (0.00)	-0.294*** (0.00)	-0.233*** (0.00)	-0.278*** (0.00)	-0.234*** (0.00)	-0.269*** (0.00)	-0.222*** (0.00)
Av_ownership					0.016 (0.44)	0.020 (0.47)	0.004 (0.86)	0.006 (0.76)
Fractional_flow_q					0.000 (0.91)	-0.000 (0.52)	0.001 (0.28)	0.000 (0.19)
Log_TNA					-0.018 (0.33)	0.003 (0.91)	0.024 (0.34)	0.000 (1.00)
Log_famsize					0.005 (0.61)	-0.004 (0.72)	-0.021* (0.08)	-0.014 (0.19)
Age					0.002 (0.15)	0.002 (0.12)	0.007*** (0.00)	0.003 (0.11)
Expense_ratio					-0.110 (0.13)	-0.207** (0.01)	-0.166* (0.06)	-0.136 (0.11)
Turnover					-0.039 (0.49)	-0.161** (0.02)	-0.111 (0.17)	-0.088 (0.17)
Constant	0.233*** (0.00)	0.215*** (0.00)	0.244*** (0.00)	0.200*** (0.00)	0.426** (0.02)	0.594*** (0.00)	0.488** (0.02)	0.494*** (0.00)
Clustered S.E.	Fund	Fund	Fund	Fund	Fund	Fund	Fund	Fund
Monthly F.E.	No	No	No	No	Yes	Yes	Yes	Yes
Observations	5752	5112	5112	5112	4584	4574	4574	4574

### 6.2. Mean reverting fund performance

The evidence is not consistent with closed funds underperforming similar open funds. Table 5 shows the average within-style performance ranks of funds that are closed to new investors and those of their comparable peers who are open. Panel A contains the mean ranks and Panel B depicts the median ranks. The ranks are from 1 to 5, where 5 indicates the best performance. The first five columns display the results for closed funds with a within-style past performance rank that corresponds to the column number. The final column shows the results for all closed funds. The ranks of closed and comparable open funds are very similar across all past performance ranks. The average difference in ranks is significant at the 5% level only in the case of funds that have a past performance rank of 3. Such closed funds have a mean contemporaneous performance rank of 2.9383 that is not statistically different from 3, whereas the comparable open funds slightly outperform with a mean performance rank of 3.0231 that is statistically different from 3 at the 1% level. The mean rank of all closed funds is 2.9907, while that of the comparable open funds is 3.0111. However, neither is statistically different from 3 at the 5% level. The statistical inferences above are also valid for the median ranks. Therefore, closed funds do not underperform their open peers. Both types of funds exhibit mean reversion in their relative performance.

**Table 5:** Average within-style performance ranks. I compare the within-style monthly net return ranks of closed funds to those of comparable open funds in the same month. I define comparable funds as those that are in the same size quintile and lagged (by one month) 12-month within-style cumulative net return rank. The table contains the average contemporaneous performance ranks of closed and open funds with a given past performance rank and the average differences. The column number indicates the past performance rank, where a higher number is indicative of better performance. The final column contains the average contemporaneous ranks of all funds used in the comparison. The null hypotheses are that the average contemporaneous ranks of both types of funds are equal to three, which is the middle rank, and that the average differences in contemporaneous ranks between closed and open funds are equal to zero. Panel A reports the mean ranks and Panel B reports the median ranks. The paired Student’s t-test is used in Panel A and the Wilcoxon signed-rank test is used in Panel B. The p-values are reported in parentheses.

Panel A: Mean ranks.						
	Lagged 12-month within-style performance rank					
	1	2	3	4	5	All
Closed	2.8614	2.9764	2.9383	3.0945	3.0934	2.9907
H_0_: Rank = 3	(0.0004)	(0.5382)	(0.1150)	(0.0217)	(0.0196)	(0.6006)
Open	2.9248	2.9794	3.0231	3.0467	3.0891	3.0111
H_0_: Rank = 3	(0.0000)	(0.0569)	(0.0036)	(0.0000)	(0.0000)	(0.0700)
Difference	-0.0634	-0.0030	-0.0848	0.0478	0.0043	-0.0204
H_0_: Diff. = 0	(0.0908)	(0.9369)	(0.0374)	(0.2467)	(0.9125)	(0.2458)
Panel B: Median ranks.						
	Lagged 12-month within-style performance rank					
	1	2	3	4	5	All
Closed	3.0000	3.0000	3.0000	3.0000	3.0000	3.0000
H_0_: Rank = 3	(0.0003)	(0.6248)	(0.0872)	(0.0077)	(0.0146)	(0.7205)
Open	2.9027	2.9716	3.0317	3.0562	3.1176	3.0159
H_0_: Rank = 3	(0.0000)	(0.0187)	(0.0021)	(0.0000)	(0.0000)	(0.0506)
Difference	-0.0625	-0.0334	-0.1000	0.0201	-0.0409	-0.0430
H_0_: Diff. = 0	(0.0505)	(0.8907)	(0.0225)	(0.1583)	(0.8880)	(0.2080)

The regression results are consistent with both closed and open funds exhibiting mean reverting abnormal returns. Table 6 contains the results of the pooled OLS regressions of fund performance on the closed dummy and past performance variables described in Section 5.2. The slope estimates of the past performance variables are positive and statistically significant at the 1% level, except in the case of the 12-month geometric average q-factor model alpha. According to the other models, an open fund with a 12-month geometric average abnormal return of 1% should expect an incremental abnormal return of between 0.062% and 0.20% in the following month. While this result is consistent with short-term abnormal fund performance persistence (as in [12]), it is evident that most of the abnormal performance disappears very quickly. Furthermore, the slope coefficients of the closed dummy and interaction terms are not statistically significant in any of the models used. In addition, propensity score matching based on the control variables in columns (5)-(8) produces results that corroborate the former and are available upon request. These results are consistent with closing a fund to new investors neither having a direct effect on performance nor affecting performance indirectly by enhancing its persistence.

**Table 6:** The marginal effect of closing to new investors on performance persistence. The table contains the output of pooled ordinary least squares (OLS) regressions with monthly observations. The dependent variable is fund performance. Return is the excess return after fund fees, Alpha-4 refers to the Carhart [11] four-factor model alpha, Alpha-5 refers to the Fama and French [20] five-factor model alpha, and Alpha-Q refers to the Hou et al. [22] q-factor model alpha. Fund alphas are estimated using the Carhart [11] methodology with the funds’ factors loading for each month estimated over trailing 12-month windows. The mean performance measures of each fund category in each month are subtracted from the measures of the corresponding funds. The demeaned measures represent abnormal percentage returns per month. Closed_dummy is a variable equal to one if the fund is closed to new investors and equal to zero otherwise. The past performance variables Lag_return, Lag_alpha_4, Lag_alpha_5 and Lag_alpha_Q are one-month-lagged fund category demeaned 12-month geometric average returns (alphas). The interaction variables Lag_return_closed, Lag_alpha_4_closed, Lag_alpha_5_closed and Lag_alpha_Q_closed are equal to the product of Closed_dummy and the respective past performance variable. All other independent variables are lagged by one month, except for the variables Expense_ratio and Turnover, which are lagged by one year. The Av_ownership variable is calculated as in [23] and represents the average ownership share (%) of a fund in the stocks of its investment portfolio. The Fractional_flow_q variable is the Sirri and Tufano [15] fractional flow (%) measure computed at the quarterly level. The p-values are reported in parentheses. *, **, and *** indicate significance at the 10%, 5%, and 1% levels, respectively.

	(1)	(2)	(3)	(4)	(5)	(6)	(7)	(8)
	Return	Alpha-4	Alpha-5	Alpha-Q	Return	Alpha-4	Alpha-5	Alpha-Q
Closed_dummy	-0.001 (0.92)	0.014 (0.47)	-0.001 (0.96)	0.018 (0.36)	-0.007 (0.65)	-0.008 (0.70)	-0.011 (0.63)	-0.002 (0.93)
Lag_return	0.087*** (0.00)				0.062*** (0.00)			
Lag_return_closed	0.001 (0.99)				-0.017 (0.68)			
Lag_alpha_4		0.083*** (0.00)				0.076*** (0.00)		
Lag_alpha_4_closed		0.064 (0.16)				0.046 (0.29)		
Lag_alpha_5			0.200*** (0.00)				0.192*** (0.00)	
Lag_alpha_5_closed			0.031 (0.43)				0.035 (0.41)	
Lag_alpha_Q				-0.018 (0.18)				-0.028* (0.06)
Lag_alpha_Q_closed				0.091 (0.11)				0.074 (0.15)
Av_ownership					-0.000*** (0.00)	-0.000*** (0.00)	-0.000*** (0.00)	-0.000*** (0.00)
Fractional_flow_q					-0.000* (0.05)	0.000 (0.97)	-0.000 (0.15)	-0.000 (0.58)
Log_TNA					-0.003 (0.26)	0.004 (0.15)	-0.001 (0.75)	0.004 (0.15)
Log_famsize					0.007*** (0.00)	0.004*** (0.00)	0.005*** (0.00)	0.004** (0.02)
Age					0.000 (0.94)	-0.000 (0.32)	0.000 (0.46)	0.000 (0.82)
Expense_ratio					-0.066*** (0.00)	-0.069*** (0.00)	-0.061*** (0.00)	-0.061*** (0.00)
Turnover					-0.016** (0.02)	-0.025*** (0.00)	-0.033*** (0.00)	-0.038*** (0.00)
Constant	-0.000 (0.98)	-0.002 (0.65)	-0.001 (0.80)	-0.001 (0.72)	0.045** (0.04)	0.034 (0.23)	0.048 (0.11)	0.045 (0.11)
Clustered S.E.	Fund	Fund	Fund	Fund	Fund	Fund	Fund	Fund
Monthly F.E.	No	No	No	No	Yes	Yes	Yes	Yes
Observations	240444	189525	189525	189525	202417	166264	166264	166264

### 6.3. Investment decision making

I also assess the investment policies of funds that close to new investors by examining their holdings around the event. Table 7 contains the average cash holdings (%), including money market instruments, of closed and comparable open funds. The first column shows the averages for the month before closure, whereas the second column displays the averages for all months during which the funds were closed. Funds that close to new investors in the next month have an average cash holding of 4.05% versus the 3.02% of similar funds that remain open, and the difference is statistically significant at the 1% level. The average cash holding of funds that are already closed is 3.36% against the 3.17% of comparable funds that are open. While the difference remains statistically significant at the 1% level during the event, its magnitude is much lower relative to before. Funds that decide to close to new investors seem to be holding too much cash and using the closure to reduce their cash holdings to more moderate levels.

**Table 7:** Average cash holdings (%). I compare the contemporaneous cash holdings (%), as reported by the CRSP Mutual Fund database, of closed funds to those of open funds in the same “Morningstar Category” (same style) and size quintile. The cash holdings include all money market instruments with maturities of one year or less. The table shows the average values and the average differences. The first column contains the average cash holding of funds that are open and will close next month and that of comparable funds that are open and will remain open next month. The second column contains the average cash holding of funds that are currently closed and the average cash holding of the comparable funds that are currently open. The t-stats are reported in parentheses.

	Month Before Closure	Months During Closure
Closed	4.05	3.36
	(11.32)	(48.82)
Open	3.02	3.17
	(41.77)	(133.82)
Difference	1.03	0.19
	(2.96)	(2.72)

**Fig 4:**
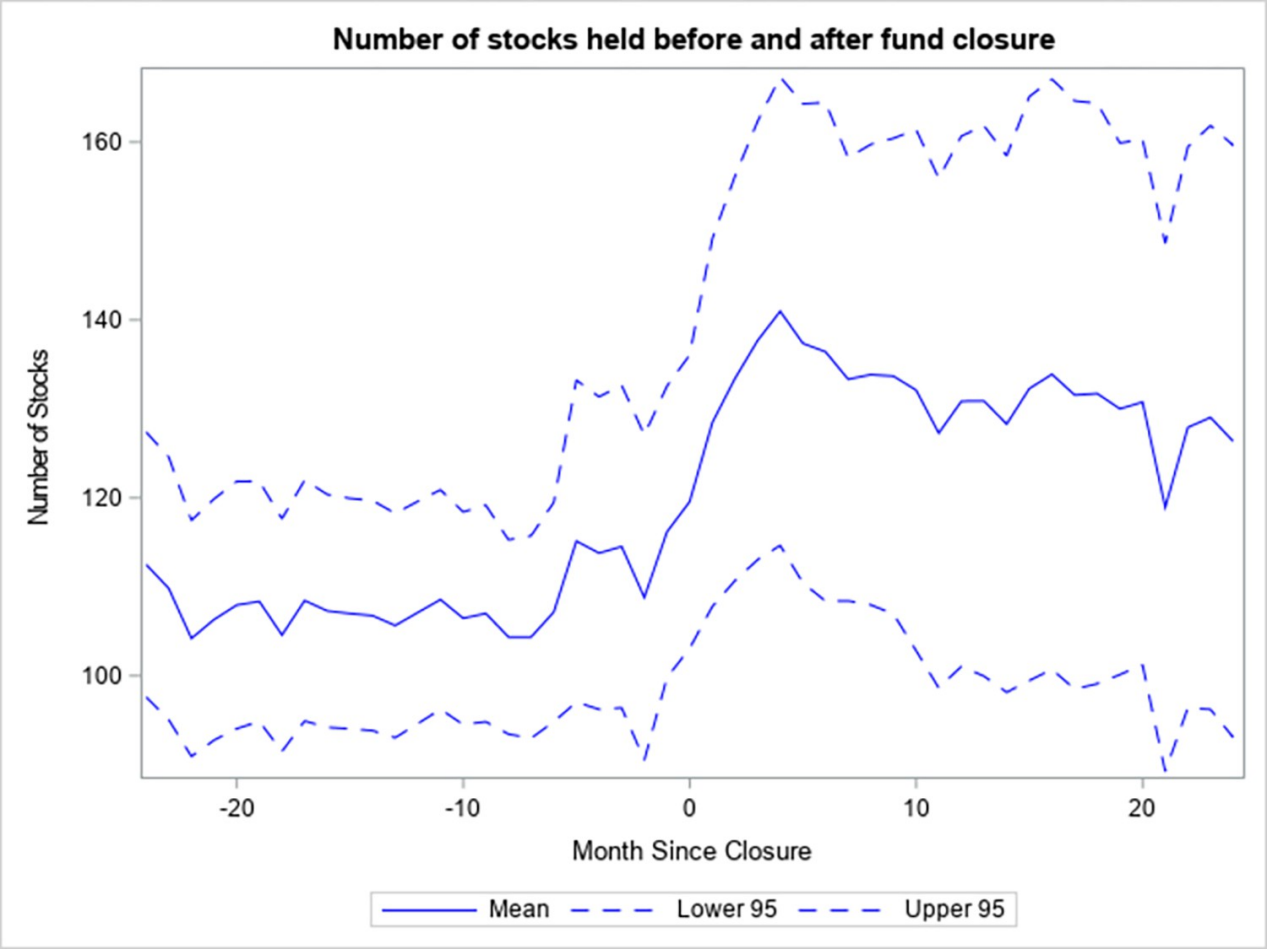
Number of stocks held before and after fund closure.

**Fig 5:**
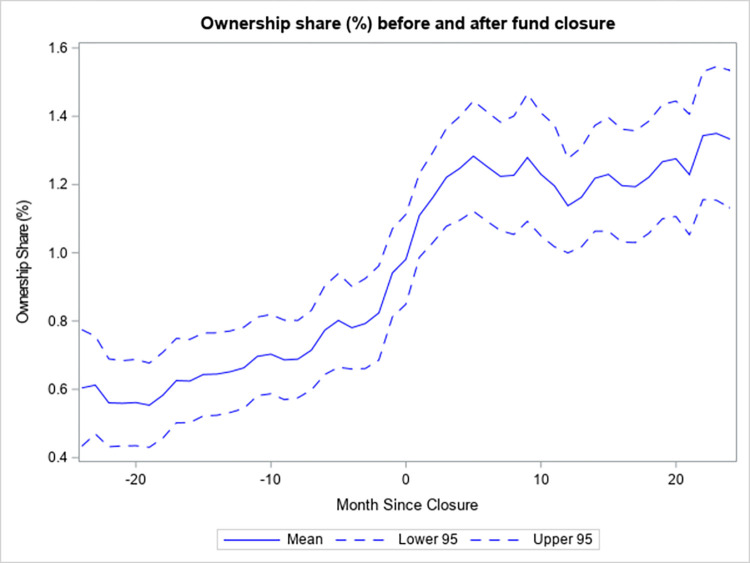
Ownership share (%) before and after fund closure.

Funds that close to new investors make additional investments with their excess cash, but also scale their existing holdings. Fig 4 shows the average number of stocks that funds which close hold in their portfolios as of between 24 months before the event and 24 months after. The average number of stocks increases in the first quarter after the event by around 20 and is relatively stable after. This is indicative of funds allocating some of their surplus cash to new investments. Fig 5 depicts the average ownership share (%) (as in [23]) around the closing event. This measure captures the average percentage of the total number of shares outstanding of the stocks in the portfolio of a fund that are held by the fund. While this measure has a clear uptrend prior to the event, its growth accelerates in the first 5 months after the event and then levels off. Overall, the measure doubles from 0.6% to 1.2%, which suggests that funds that close to new investors aggressively scale existing positions in order to reduce their cash holdings. The resulting concentration in ownership may deter funds from unwinding unwanted positions due to liquidity constraints in the form of price impact. Given that Simutin [24] provides evidence that active equity mutual funds with abnormally high cash holdings subsequently outperform their peers with low abnormal cash holdings, the funds that closed to new investors may have performed better had they retained their high pre-closure cash holdings after the event.

Fig 4 displays the number of stocks held by funds that close to new investors from 24 months before the closure to 24 months after. Mean is the average number of stocks as of a certain number of months relative to the event. Lower 95 is the lower bound of the 95% confidence interval for the mean and Upper 95 is the upper bound. The number of stocks is counted from the fund holdings data of the CRSP Mutual Fund database. In months, in which the fund holdings are missing, the most recently available fund holdings are used, provided that they are not older than one quarter.

Fig 5 portrays the average ownership share (in percent), as in (22), of funds that close to new investors from 24 months before the closure to 24 months after. The measure is the fund portfolio level average of the fraction of the total number of shares of a stock outstanding that are held by the fund. Mean is the average of this measure as of a certain number of months relative to the event. Lower 95 is the lower bound of the 95% confidence interval for the mean and Upper 95 is the upper bound. The ownership share is calculated from the fund holdings data of the CRSP Mutual Fund database. In months, in which the fund holdings are missing, the most recently available fund holdings are used, provided that they are not older than one quarter.

### 6.4. Mean reverting pre-closure fund portfolio performance

The evidence is not consistent with funds changing their investment strategies following their closure to new investors enough for the changes to have a substantial impact on their risk-adjusted performance. Table 8 contains the performance results of the “ghost” portfolios described in Section 5.3 and the performance of actual closed funds before fees (see S2 Appendix for factor exposures). Panel A contains the performance of the “ghost” portfolios without rebalancing. The funds have average excess return that is lower than that of the portfolios by 0.04% per month, and the difference is not statistically significant at the 5% level. Furthermore, the average differences in alphas are of negligible sizes and are not statistically significant. In addition, the average alphas of both the closed funds and the “ghost” portfolios are not statistically significant. Panel B shows the results for the “ghost” portfolios with quarterly rebalancing. These portfolios perform considerably better relative to the portfolios without rebalancing. The former beat the closed funds in terms of excess return by 0.17% per month on average, which is statistically significant at the 1% level. Likewise, the rebalanced “ghost” portfolios substantially outperform the funds in terms of alpha. This suggests that the closed funds may not be rebalancing their actual portfolios enough. Furthermore, Panel C depicts that funds which close to new investors have on average 72.72% (56.98%) of their portfolios invested in “ghost” portfolio stocks as of 12 (24) months after “ghost” portfolio formation. Similarly, on average 66.45% (51.49%) of the funds’ portfolios were invested in those stocks as of 12 (24) months before formation. Therefore, closed funds do not make impactful investment decisions around the time of closing and as a result suffer from the mean reversion in the performance of the stocks that had previously outperformed.

**Table 8:** Comparison of actual funds to their pre-closure (“ghost”) portfolios after closure. I form (“ghost”) portfolios, each consisting of the same stocks that a fund held immediately before closing to new investors. The initial portfolio weights are the same as the weights of the actual portfolio of the fund at the time. This method resembles the approach of Lapatto and Puttonen [10]. Each portfolio is formed with the corresponding fund holdings as of the last available report date prior to closure, given that it is not more than a quarter before the event. A fund must have been open to investors for at least 24 consecutive months prior to the closing event in order for its “ghost” portfolio to be formed. The portfolio is held passively for 24 months after the event. Portfolio alphas are calculated at the individual portfolio level using time series regressions of the monthly portfolio excess returns against the relevant factors. Fund alphas are calculated at the individual fund level by regressing the fund excess returns before fees against the corresponding factors. The post-closure gross returns of a fund must be available for no less than 12 months. The table contains the average monthly performance measures (%) of the funds and the portfolios as well as the average monthly pairwise differences in performance (%). Panel A shows the results when the “ghost” portfolios are not rebalanced, whereas Panel B portrays the results when the portfolios are rebalanced quarterly. The t-stats are reported in parentheses. Panel C contains the average percentage of the actual fund portfolio that is invested in the stocks of the corresponding “ghost” portfolio as of a given number of months relative to “ghost” portfolio formation.

Panel A: Performance comparison under no rebalancing of “ghost” portfolio.				
	Excess Return	Carhart Alpha	F&F Five-Factor Alpha	Q-Factor Alpha
Fund	0.55	-0.02	0.01	0.02
	(9.57)	(-0.94)	(0.48)	(0.71)
Ghost Portfolio	0.59	-0.02	0.00	0.01
	(10.11)	(-0.93)	(-0.08)	(0.44)
Difference	-0.04	0.00	0.01	0.01
	(-1.76)	(-0.04)	(0.65)	(0.32)
Panel B: Performance comparison under quarterly rebalancing of “ghost” portfolio.				
	Excess Return	Carhart Alpha	F&F Five-Factor Alpha	Q-Factor Alpha
Fund	0.55	-0.02	0.01	0.02
	(9.57)	(-0.94)	(0.48)	(0.71)
Ghost Portfolio	0.72	0.08	0.19	0.17
	(13.12)	(3.02)	(5.99)	(6.40)
Difference	-0.17	-0.10	-0.18	-0.15
	(-6.60)	(-4.44)	(-5.78)	(-5.74)

Panel C: Percentage of fund portfolio invested in “ghost” portfolio stocks.								
	Months relative to “ghost” portfolio formation							
	-24	-18	-12	-6	6	12	18	24
Percentage Invested	51.49	56.95	66.45	79.26	82.71	72.72	63.60	56.98
Number of Closing Events	327	370	404	444	309	267	266	255

### 6.5. Ineffective size preservation

**Fig 6:**
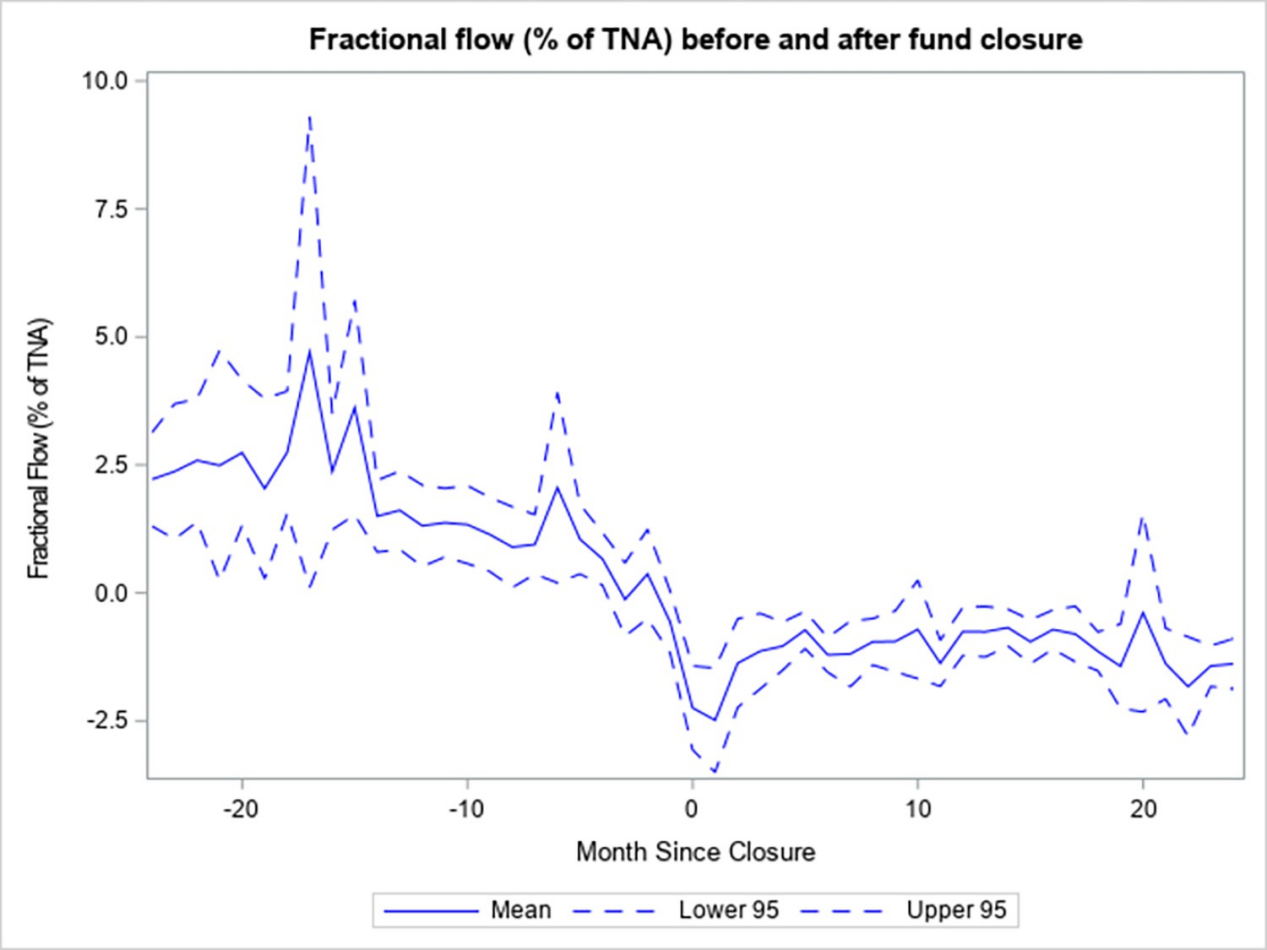
Fractional flow (% of TNA) before and after fund closure.

Previous studies show that fund flows are abnormally high before the closure to new investors, and this is not the case after the event. Fig 6 depicts the average Sirri and Tufano [15] fractional flow (%) from 24 months before the event to 24 months after. The figure is consistent with the results of prior studies. Funds that close have average fractional flows of between 2% and 3% of their assets under management (AUM) in most of the months in the two years just before closing and have negative fractional flows of about -1% of their AUM during most of the months in the two years immediately following the closing.

Fig 6 depicts the fractional flow (in percent of total net assets), as in (14), of funds that close to new investors from 24 months before the closure to 24 months after. The measure is the part of the monthly growth rate of a fund’s total net assets which is in excess of the net monthly return of the fund. Mean is the average fractional flow as of a certain number of months relative to the event. Lower 95 is the lower bound of the 95% confidence interval for the mean and Upper 95 is the upper bound. The total net assets and net returns of the funds are obtained from the CRSP Mutual Fund database.

I also analyze New Sales (%) and Redemptions (%) separately and compare the flows of closed funds to those of similar open funds in order to test my defensive strategy hypothesis. Table 9 portrays the average New Sales (%) (Panel A), Redemptions (%) (Panel B) and Net Cash Flow (%) (Panel C) of funds that close to new investors and comparable open funds. Each of the first five columns shows the averages for funds belonging to the within-style past performance rank that corresponds to the column number, where a higher number indicates better past performance. The averages for all funds are in the final column. Average New Sales (%) of closed funds are lower across all ranks, and the difference is statistically significant at the 1% level, with the exception of the 4^th^ rank. Overall, closed funds have average New Sales of 1.74% per month, which is lower relative to the 2.82% of comparable open funds. Therefore, New Sales to existing investors of closed funds are not high enough to offset their loss of New Sales to new investors.

**Table 9:** Fund flow measures. I compare the flows of closed funds to those of comparable open funds in the same month. I define comparable funds as those that are in the same size quintile and lagged (by one month) 12-month within-style cumulative net return rank. The table contains the average flows of closed and open funds with a given past performance rank and the average differences. The column number indicates the past performance rank, where a higher number is indicative of better performance. The final column contains the average flows of all funds used in the comparison. The flow measures are New Sales (%) in Panel A, Redemptions (%) in Panel B and their difference—Net Cash Flow (%) in Panel C. All flow measures are equal to the corresponding dollar amounts, as reported by Morningstar Direct, divided by the fund size in the previous month, as in [15], and multiplied by 100. The t-stats are reported in parentheses.

Panel A: New Sales (%).						
	Lagged 12-month within-style performance rank					
	1	2	3	4	5	All
Closed	1.19	1.37	1.56	2.27	2.36	1.74
	(15.89)	(24.75)	(27.43)	(6.96)	(7.43)	(18.94)
Open	1.93	2.26	2.58	2.86	4.53	2.82
	(41.17)	(40.24)	(39.97)	(40.49)	(54.68)	(84.66)
Difference	-0.74	-0.89	-1.02	-0.59	-2.17	-1.08
	(-9.54)	(-11.41)	(-11.48)	(-1.77)	(-6.98)	(-11.57)
Panel B: Redemptions (%).						
	Lagged 12-month within-style performance rank					
	1	2	3	4	5	All
Closed	4.08	3.57	3.54	3.05	2.45	3.35
	(23.96)	(22.27)	(13.24)	(17.78)	(14.99)	(39.44)
Open	3.36	2.85	2.60	2.45	2.45	2.76
	(93.11)	(85.05)	(71.33)	(78.14)	(84.57)	(169.57)
Difference	0.72	0.72	0.94	0.60	0.00	0.59
	(4.37)	(4.55)	(3.53)	(3.60)	(0.00)	(7.13)
Panel C: Net Cash Flow (%).						
	Lagged 12-month within-style performance rank					
	1	2	3	4	5	All
Closed	-2.89	-2.20	-1.97	-0.78	-0.09	-1.62
	(-16.61)	(-13.65)	(-7.26)	(-2.30)	(-0.31)	(-14.09)
Open	-1.43	-0.59	-0.02	0.41	2.08	0.06
	(-27.64)	(-10.57)	(-0.30)	(5.92)	(26.10)	(1.61)
Difference	-1.46	-1.61	-1.95	-1.19	-2.17	-1.68
	(-8.52)	(-9.45)	(-6.87)	(-3.43)	(-7.43)	(-14.57)

While closing a fund to new investors is detrimental to New Sales, it might be beneficial by reducing Redemptions. This is clearly not the case, as average Redemptions of closed funds are higher relative to those of similar open funds for all past performance ranks, except for the top one, in which both averages are 2.45%. The other differences are statistically significant at the 1% level. Overall, closed funds have higher average monthly Redemptions of 3.35% relative to the 2.76% of comparable open funds. Somewhat surprisingly, investors are more willing to take money out of closed funds, despite their exclusivity. One possible explanation is that the closed funds obtained very performance-sensitive investors during the period before closing when they outperformed.

Net Cash Flow is simply the difference between New Sales and Redemptions. Therefore, it is not surprising, given the results above, that funds which close to new investors have lower average Net Cash Flow across all past performance ranks. Notably, closed funds have an average Net Cash Flow of -1.62%, while comparable open funds have an average Net Cash Flow of 0.06%. Therefore, closing a fund to new investors is not an effective strategy to preserve fund size in the face of deteriorating performance. Furthermore, given that the open funds used as the counterfactual received very low average flows, closing a fund may not be necessary in most cases to avoid unwanted large future flows.

## 7. Conclusion

I study the causes and consequences of the decision of active US equity mutual funds to close to new investors. I start by analyzing results of the prior literature, which has focused on performance before and after closure to new investors. I confirm the key findings of several previous studies—mutual funds exhibit superior performance and receive large flows from investors prior to closing and do not maintain their superior performance following the closure. These findings motivated further investigation, since a comprehensive explanation was lacking in the literature.

I argue that the performance reversal is due to a combination of mean reversal of the returns of the stocks held by the funds and liquidity constraints. Specifically, I show that the gross alpha of a fund after closure does not substantially differ from that of a hypothetical buy-and-hold “ghost” portfolio consisting of the same stocks the fund held just before closing to new investors. Furthermore, the actual portfolio allocations of the fund after closing resemble those of the “ghost” portfolio. This is evidence that the funds largely continue on a preset course and do not change their investment strategies substantially after closing. Therefore, the deterioration of the performance of those funds must be due to the mean reverting returns of stocks that have recently outperformed. I also show that a simple quarterly rebalancing of the “ghost” portfolio improves performance, suggesting that the funds may be reluctant to sell their winners and buy their losers. This could be driven by a behavioral bias or liquidity constraints in the form of price impact, since these funds are very large relative to the average fund.

I also show that closed funds do not underperform open funds after controlling for fund size and within-style past performance rank. The two sets of funds exhibit similar strong mean reversion of their within-style performance ranks. This shows that the closing decision in itself is not consequential to performance, which is consistent with previous research.

I also investigate the investment policies of the funds around the closure events in more detail. I find that the funds that close hold a larger percentage of cash prior to closing relative to other funds in the same style and size quintile that remain open. This difference almost disappears after closing, suggesting that the funds that close use their additional cash to make investment decisions. While there is some evidence that they increase the number of stocks in their portfolios, the average ownership share in the stocks of the funds’ portfolios continues to increase after closure. This result is indicative of liquidity constraints such as price impact becoming more severe due to the allocation of accumulated cash despite the refusal to accept additional cash from new investors.

Finally, I reject the possibility of funds closing to new investors as a defensive strategy to preserve existing assets under management by introducing exclusivity to their current investors. After controlling for fund size and within-style past performance, fractional flow is 1.68% per month lower for closed funds as opposed to open funds, of which 1.08% is due to lower new sales, and the remaining 0.60% is due to higher redemptions. Somewhat surprisingly, redemptions are higher for closed funds, except in the top performance quintile, where they are on par with those of similar open funds. This is indicative of closed funds having highly performance-sensitive investors. Furthermore, the control group of open funds has an average fractional flow of just 0.06% per month, which suggests that the funds which closed would not have continued to receive large flows going forward, even had they remained open to new investors. This raises further doubt regarding the necessity of closing a fund to new investors.

There are some important policy implications of these findings. First, the procedure required to close a mutual fund to new investors should be regulated in a comprehensive manner. Mutual funds should be obliged to formally justify their decision to both existing and potential investors and provide a detailed plan on how they will manage their liquidity during the closure period. Furthermore, the funds should be required to disclose a provisional reopening date, so that potential new investors can wait for the funds to reopen, instead of looking for potentially more expensive substitutes for the closed funds in their fund families. Overall, increased transparency would enable investors to make optimal decisions, and ensure that mutual funds close to new investors only for well-justified reasons.

Finally, I propose some avenues for future research. We still do not have a comprehensive picture of the complex motivations that drive mutual fund managers to close their funds to new investors. One option to expand our knowledge base is to study how managerial characteristics, such as age, gender and tenure, that are linked to certain behavioral patterns, impact both the propensity to close and the subsequent outcomes. Another alternative is to conduct a wide-scale survey of fund managers that have closed their funds to new investors, in order to identify potentially hidden motivations. For this survey to be effective, anonymity should be guaranteed to the participating fund managers.

## Supporting information

S1 Appendix Performance of small cap growth funds after closing to new investors. (DOCX)

S2 Appendix Factor exposures of closed funds and “ghost” portfolios. (DOCX)
