# Supplementary material for: The paradox of closing mutual funds to new investors
Source: PLoS One. 2023 Sep 14;18(9):e0290254. doi: 10.1371/journal.pone.0290254 (PMC10501617; doi:10.1371/journal.pone.0290254)
Supplement: S1 Appendix — (DOCX) [file pone.0290254.s001.docx]

**Appendix A. Performance of small cap growth funds after closing to new investors**

Closing a fund to new investors imposes a restriction on the growth of the size of the fund. While a growing fund size may have an adverse impact on the performance of all funds, the magnitude of the effect need not be uniform across different types of funds. Diseconomies of scale should be a bigger concern for small cap growth funds, because they are facing the most severe liquidity issues (as according to Chen et al. [1]). Therefore, closing such funds to new investors is most likely to be beneficial in terms of superior performance preservation. I run additional tests to determine if closing a fund is effective for this subset of funds.

Table A1 contains the results of the regressions described in Section 5.1 and tabulated in Table 4 with the addition of a small growth dummy variable that is equal to one if the Morningstar Category of a fund is “Small Growth” and zero otherwise and an interaction variable that is equal to the product of the closed dummy and the small growth dummy. The magnitude of the slope coefficient of the small growth dummy is positive and statistically significant at the 5% level in most specifications indicating that small cap growth funds performed particularly well relative to other funds prior to closing. These funds earned an additional abnormal return as high as 21.4 basis points per month in the 24-month period before closing to new investors according to the estimate in column 8 of Panel C. However, their subsequent additional decline in performance relative to other funds was at least as pronounced. Small cap growth funds had an additional abnormal return reduction of 29.4 basis points per month in the 24 months after closing according to the slope coefficient estimates of the interaction variable in columns 6 and 7 of Panel C. Overall, the evidence is not consistent with the decision to close a small cap growth fund facilitating the preservation of superior performance.

Table A1: The marginal effect of closing small-cap growth funds to new investors on risk-adjusted performance

The table contains the output of pooled ordinary least squares (OLS) regressions with monthly observations. Only funds that close to new investors are included. The dependent variable is fund performance. Return is the excess return after fund fees, Alpha-4 refers to the Carhart [2] four-factor model alpha, Alpha-5 refers to the Fama et al. [3] five-factor model alpha, and Alpha-Q refers to the Hou et al. [4] q-factor model alpha. Fund alphas are estimated using the Carhart [2] methodology with the funds’ factors loading for each month estimated over trailing 12-month windows. The mean performance measures of each fund category in each month are subtracted from the measures of the corresponding funds. The demeaned measures represent abnormal percentage returns per month. The dummy variable Closed (Small_growth) is equal to one if the fund is closed to new investors (a small cap growth fund) and equal to zero otherwise. The interaction variable Small_growth_closed is equal to the product of Closed and Small_growth. All other independent variables are lagged by one month, except for the variables Expense_ratio and Turnover, which are lagged by one year. The Av_ownership variable is calculated as in [5]. The Fractional_flow_q variable is the Sirri et al. [6] fractional flow (%) measure computed at the quarterly level. The observations in Panel A represent the entire within sample period histories of funds that close to new investors up to the points at which they reopen. The observations in Panel B are further restricted to be within 24 months of the closing events. I additionally restrict the sample in Panel C to funds that have return data for each of the 12 months immediately before and after their closings. The p-values are reported in parentheses. *, **, and *** indicate significance at the 10%, 5%, and 1% levels, respectively.

Panel A: Pooled OLS regressions with all observations prior to the funds reopening

|  | (1) | (2) | (3) | (4) | (5) | (6) | (7) | (8) |
| --- | --- | --- | --- | --- | --- | --- | --- | --- |
|  | Return | Alpha-4 | Alpha-5 | Alpha-Q | Return | Alpha-4 | Alpha-5 | Alpha-Q |
| Closed | -0.035 (0.18) | -0.027 (0.39) | -0.034 (0.34) | -0.039 (0.18) | -0.057^*^ (0.08) | -0.082^**^ (0.04) | -0.057 (0.18) | -0.059 (0.11) |
| Small_growth | 0.149^***^ (0.00) | 0.155^***^ (0.00) | 0.194^***^ (0.00) | 0.146^***^ (0.00) | 0.137^***^ (0.00) | 0.166^***^ (0.00) | 0.153^***^ (0.01) | 0.149^***^ (0.00) |
| Small_growth_closed | -0.153^***^ (0.00) | -0.181^***^ (0.00) | -0.215^***^ (0.00) | -0.149^**^ (0.01) | -0.123^**^ (0.04) | -0.147^**^ (0.03) | -0.166^**^ (0.02) | -0.132^**^ (0.04) |
| Av_ownership |  |  |  |  | 0.006 (0.57) | -0.000 (0.99) | 0.029^*^ (0.09) | 0.013 (0.37) |
| Fractional_flow_q |  |  |  |  | 0.001 (0.16) | 0.001 (0.18) | 0.002^**^ (0.05) | 0.001^*^ (0.07) |
| Log_TNA |  |  |  |  | 0.014 (0.10) | 0.031^***^ (0.00) | 0.009 (0.44) | 0.012 (0.20) |
| Log_famsize |  |  |  |  | 0.006 (0.11) | 0.001 (0.81) | -0.001 (0.90) | -0.002 (0.60) |
| Age |  |  |  |  | -0.000 (0.94) | -0.001 (0.50) | 0.002^*^ (0.09) | 0.000 (0.83) |
| Expense_ratio |  |  |  |  | -0.110^***^ (0.01) | -0.129^***^ (0.01) | -0.064 (0.23) | -0.082^*^ (0.08) |
| Turnover |  |  |  |  | 0.016 (0.54) | -0.038 (0.26) | -0.000 (0.99) | -0.028 (0.35) |
| Constant | 0.037^**^ (0.03) | 0.037^*^ (0.07) | 0.047^*^ (0.05) | 0.047^**^ (0.02) | -0.063 (0.46) | -0.020 (0.85) | -0.019 (0.86) | 0.032 (0.74) |
| Clustered S.E. | Fund | Fund | Fund | Fund | Fund | Fund | Fund | Fund |
| Monthly F.E. | No | No | No | No | Yes | Yes | Yes | Yes |
| Observations | 23084 | 17994 | 17994 | 17994 | 15605 | 15558 | 15558 | 15558 |

Table A1: The marginal effect of closing small-cap growth funds to new investors on risk-adjusted performance (cont.)

Panel B: Pooled OLS regressions with observations that are within 24 months of the closings

|  | (1) | (2) | (3) | (4) | (5) | (6) | (7) | (8) |
| --- | --- | --- | --- | --- | --- | --- | --- | --- |
|  | Return | Alpha-4 | Alpha-5 | Alpha-Q | Return | Alpha-4 | Alpha-5 | Alpha-Q |
| Closed | -0.056^*^ (0.06) | -0.030 (0.41) | -0.047 (0.30) | -0.072^**^ (0.04) | -0.076^**^ (0.05) | -0.081^*^ (0.06) | -0.063 (0.24) | -0.091^**^ (0.02) |
| Small_growth | 0.172^***^ (0.00) | 0.145^**^ (0.02) | 0.197^***^ (0.00) | 0.155^***^ (0.01) | 0.140^***^ (0.01) | 0.140^**^ (0.03) | 0.156^**^ (0.01) | 0.131^**^ (0.03) |
| Small_growth_closed | -0.161^***^ (0.01) | -0.189^**^ (0.01) | -0.252^***^ (0.01) | -0.146^**^ (0.05) | -0.109 (0.13) | -0.162^*^ (0.05) | -0.230^***^ (0.01) | -0.123 (0.12) |
| Av_ownership |  |  |  |  | 0.001 (0.92) | 0.016 (0.45) | 0.043^*^ (0.07) | 0.024 (0.16) |
| Fractional_flow_q |  |  |  |  | 0.001 (0.15) | 0.001 (0.21) | 0.002^*^ (0.06) | 0.002^*^ (0.06) |
| Log_TNA |  |  |  |  | 0.029^**^ (0.01) | 0.037^***^ (0.01) | 0.007 (0.65) | 0.014 (0.25) |
| Log_famsize |  |  |  |  | 0.001 (0.84) | -0.001 (0.92) | 0.003 (0.70) | -0.001 (0.91) |
| Age |  |  |  |  | -0.001 (0.50) | -0.001 (0.39) | 0.001 (0.44) | -0.001 (0.62) |
| Expense_ratio |  |  |  |  | -0.085^*^ (0.08) | -0.073 (0.23) | 0.009 (0.91) | -0.029 (0.61) |
| Turnover |  |  |  |  | 0.011 (0.72) | -0.020 (0.62) | -0.039 (0.40) | -0.029 (0.45) |
| Constant | 0.042^**^ (0.04) | 0.044^*^ (0.07) | 0.061^**^ (0.03) | 0.060^***^ (0.01) | -0.132 (0.21) | -0.103 (0.40) | -0.064 (0.66) | -0.060 (0.58) |
| Clustered S.E. | Fund | Fund | Fund | Fund | Fund | Fund | Fund | Fund |
| Monthly F.E. | No | No | No | No | Yes | Yes | Yes | Yes |
| Observations | 14137 | 11204 | 11204 | 11204 | 9717 | 9674 | 9674 | 9674 |

Table A1: The marginal effect of closing small-cap growth funds to new investors on risk-adjusted performance (cont.)

Panel C: Pooled OLS regressions with observations that are within 24 months of the closings (reduced sample)

|  | (1) | (2) | (3) | (4) | (5) | (6) | (7) | (8) |
| --- | --- | --- | --- | --- | --- | --- | --- | --- |
|  | Return | Alpha-4 | Alpha-5 | Alpha-Q | Return | Alpha-4 | Alpha-5 | Alpha-Q |
| Closed | -0.257^***^ (0.00) | -0.158^***^ (0.00) | -0.219^***^ (0.00) | -0.191^***^ (0.00) | -0.247^***^ (0.00) | -0.157^***^ (0.00) | -0.191^***^ (0.00) | -0.172^***^ (0.00) |
| Small_growth | 0.051 (0.45) | 0.118 (0.13) | 0.150^*^ (0.08) | 0.165^**^ (0.03) | 0.058 (0.45) | 0.206^***^ (0.01) | 0.169^*^ (0.05) | 0.214^**^ (0.01) |
| Small_growth_closed | -0.097 (0.26) | -0.230^***^ (0.01) | -0.280^**^ (0.01) | -0.157^*^ (0.08) | -0.115 (0.21) | -0.294^***^ (0.00) | -0.294^***^ (0.00) | -0.201^**^ (0.03) |
| Av_ownership |  |  |  |  | 0.016 (0.45) | 0.018 (0.51) | 0.003 (0.90) | 0.004 (0.86) |
| Fractional_flow_q |  |  |  |  | 0.000 (0.89) | -0.000 (0.59) | 0.001 (0.26) | 0.001 (0.16) |
| Log_TNA |  |  |  |  | -0.019 (0.34) | 0.005 (0.84) | 0.022 (0.40) | 0.008 (0.69) |
| Log_famsize |  |  |  |  | 0.005 (0.58) | -0.004 (0.73) | -0.020^*^ (0.09) | -0.014 (0.16) |
| Age |  |  |  |  | 0.002 (0.19) | 0.002 (0.29) | 0.007^***^ (0.00) | 0.002 (0.44) |
| Expense_ratio |  |  |  |  | -0.110 (0.14) | -0.219^***^ (0.01) | -0.171^*^ (0.07) | -0.160^*^ (0.06) |
| Turnover |  |  |  |  | -0.040 (0.49) | -0.168^**^ (0.01) | -0.116 (0.15) | -0.096 (0.13) |
| Constant | 0.219^***^ (0.00) | 0.184^***^ (0.00) | 0.205^***^ (0.00) | 0.157^***^ (0.00) | 0.417^**^ (0.02) | 0.554^***^ (0.00) | 0.459^**^ (0.03) | 0.445^***^ (0.01) |
| Clustered S.E. | Fund | Fund | Fund | Fund | Fund | Fund | Fund | Fund |
| Monthly F.E. | No | No | No | No | Yes | Yes | Yes | Yes |
| Observations | 5752 | 5112 | 5112 | 5112 | 4584 | 4574 | 4574 | 4574 |

**References**

1. Chen J, Hong H, Huang M, Kubik JD. Does fund size erode mutual fund performance? The role of liquidity and organization. Am Econ Rev. 2004;94(5):1276-1302.
2. Carhart MM. On persistence in mutual fund performance. J Finance. 1997;52(1):57-82.
3. Fama EF, French KR. A five-factor asset pricing model. J Financ Econ. 2015;116(1):1-22.
4. Hou K, Xue C, Zhang L. Digesting anomalies: An investment approach. Rev Financ Stud. 2015;28(3):650-705.
5. Pollet JM, Wilson M. How does size affect mutual fund behavior? J Finance. 2008;63(6):2941-2969.
6. Sirri ER, Tufano P. Costly search and mutual fund flows. J Finance. 1998;53(5):1589-1622.
