# Supplementary material for: The paradox of closing mutual funds to new investors
Source: PLoS One. 2023 Sep 14;18(9):e0290254. doi: 10.1371/journal.pone.0290254 (PMC10501617; doi:10.1371/journal.pone.0290254)
Supplement: S2 Appendix — (DOCX) [file pone.0290254.s002.docx]

**Appendix B. Factor exposures of closed funds and “ghost” portfolios**

Table B1 shows the average factor exposures (betas) of the “ghost” portfolios, described in Section 5.3, and the respective closed funds. The “ghost” portfolios in Panels A, C and E are not rebalanced. The closed funds have strongly positive loadings on the size factor (SMB and ME) and slightly negative loadings on the value factor (HML), which is consistent with “Small Growth” being the most common categorization of funds that close to new investors (see Table 3). In the absence of the value factor in the model, there is a negative loading on the profitability factor (ROE) of a similar magnitude. Furthermore, there is a robust negative loading on the investment factor (CMA and IA), suggesting that the funds invest in companies with aggressive investment policies. The exposures of the closed funds to the factors are broadly similar to those of the corresponding “ghost” portfolios and are in most cases slightly lower, which implies that the funds mildly reduce systematic risk after closing to new investors. Regularly rebalancing the “ghost” portfolios, as in Panels B, D and F, increases their loadings on the size and value factors, reduces their loadings on the momentum and profitability factors and has an unclear effect on their loadings on the market and investment factors.

Table B1: Comparison of actual fund betas and “ghost” portfolio betas after closure

I form (“ghost”) portfolios, each consisting of the same stocks that a fund held immediately before closing to new investors. The initial portfolio weights are the same as the weights of the actual portfolio of the fund at the time. This method resembles the approach of Lapatto et al. [1]. Each portfolio is formed with the corresponding fund holdings as of the last available report date prior to closure, given that it is not more than a quarter before the event. A fund must have been open to investors for at least 24 consecutive months prior to the closing event in order for its “ghost” portfolio to be formed. The portfolio is held passively for 24 months after the event. Portfolio factor exposures (betas) are calculated at the individual portfolio level using time series regressions of the monthly portfolio excess returns against the relevant factors. Fund betas are calculated at the individual fund level by regressing the fund excess returns before fees against the corresponding factors. The post-closure gross returns of a fund must be available for no less than 12 months. The table contains the average betas of the funds and the portfolios as well as the average pairwise differences in betas. The models used are the Carhart [2] four-factor model (Panels A and B), the Fama et al. [3] five-factor model (Panels C and D) and the Hou et al. [4] q-factor model (Panels E and F). Panels A, C and E show the results when the “ghost” portfolios are not rebalanced, whereas Panels B, D and F portray the results when the portfolios are rebalanced quarterly. The t-stats are reported in parentheses.

Panel A: Carhart [2] four-factor model betas comparison under no rebalancing of “ghost” portfolio

|  | MKTRF | SMB | HML | UMD |
| --- | --- | --- | --- | --- |
| Fund | 0.99 | 0.47 | -0.05 | 0.00 |
|  | (77.99) | (20.93) | (-2.61) | (-0.14) |
| Ghost Portfolio | 1.05 | 0.49 | -0.03 | 0.00 |
|  | (97.72) | (22.22) | (-1.25) | (0.05) |
| Difference | -0.06 | -0.02 | -0.02 | 0.00 |
|  | (-5.80) | (-2.04) | (-2.58) | (-0.26) |

Panel B: Carhart [2] four-factor model betas comparison under quarterly rebalancing of “ghost” portfolio

|  | MKTRF | SMB | HML | UMD |
| --- | --- | --- | --- | --- |
| Fund | 0.99 | 0.47 | -0.05 | 0.00 |
|  | (77.99) | (20.93) | (-2.61) | (-0.14) |
| Ghost Portfolio | 1.04 | 0.53 | -0.01 | -0.10 |
|  | (96.66) | (22.26) | (-0.46) | (-8.22) |
| Difference | -0.05 | -0.06 | -0.04 | 0.10 |
|  | (-5.21) | (-4.94) | (-4.02) | (9.48) |

Table B1: Comparison of actual fund betas and “ghost” portfolio betas after closure (cont.)

Panel C: Fama and French [3] five-factor model betas comparison under no rebalancing of “ghost” portfolio

|  | MKTRF | SMB | HML | RMW | CMA |
| --- | --- | --- | --- | --- | --- |
| Fund | 0.97 | 0.47 | -0.05 | -0.04 | -0.19 |
|  | (75.96) | (20.22) | (-2.01) | (-1.52) | (-7.36) |
| Ghost Portfolio | 1.03 | 0.50 | -0.04 | 0.00 | -0.18 |
|  | (88.31) | (21.46) | (-1.79) | (0.08) | (-6.93) |
| Difference | -0.06 | -0.03 | -0.01 | -0.04 | -0.01 |
|  | (-5.43) | (-2.92) | (-0.47) | (-2.75) | (-0.35) |

Panel D: Fama and French [3] five-factor model betas comparison under quarterly rebalancing of “ghost” portfolio

|  | MKTRF | SMB | HML | RMW | CMA |
| --- | --- | --- | --- | --- | --- |
| Fund | 0.97 | 0.47 | -0.05 | -0.04 | -0.19 |
|  | (75.96) | (20.22) | (-2.01) | (-1.52) | (-7.36) |
| Ghost Portfolio | 1.05 | 0.55 | 0.01 | -0.03 | -0.19 |
|  | (82.96) | (21.38) | (0.53) | (-1.09) | (-6.53) |
| Difference | -0.08 | -0.08 | -0.06 | -0.01 | 0.00 |
|  | (-7.05) | (-6.17) | (-4.28) | (-0.35) | (-0.20) |

Panel E: Hou et al. [4] q-factor model betas comparison under no rebalancing of “ghost” portfolio

|  | MKTRF | ME | IA | ROE |
| --- | --- | --- | --- | --- |
| Fund | 0.95 | 0.43 | -0.20 | -0.07 |
|  | (76.72) | (19.53) | (-7.05) | (-3.90) |
| Ghost Portfolio | 1.01 | 0.45 | -0.18 | -0.05 |
|  | (99.12) | (21.30) | (-5.83) | (-2.54) |
| Difference | -0.06 | -0.02 | -0.02 | -0.02 |
|  | (-6.21) | (-2.17) | (-1.81) | (-1.42) |

Panel F: Hou et al. [4] q-factor model betas comparison under quarterly rebalancing of “ghost” portfolio

|  | MKTRF | ME | IA | ROE |
| --- | --- | --- | --- | --- |
| Fund | 0.95 | 0.43 | -0.20 | -0.07 |
|  | (76.72) | (19.53) | (-7.05) | (-3.90) |
| Ghost Portfolio | 1.00 | 0.47 | -0.13 | -0.22 |
|  | (94.02) | (20.94) | (-3.86) | (-9.27) |
| Difference | -0.05 | -0.04 | -0.07 | 0.15 |
|  | (-5.00) | (-3.07) | (-4.71) | (7.44) |

**References**

1. Lapatto A, Puttonen V. Life after death: acquired fund performance. Manag Finance. 2018;44(3):389-402.
2. Carhart MM. On persistence in mutual fund performance. J Finance. 1997;52(1):57-82.
3. Fama EF, French KR. A five-factor asset pricing model. J Financ Econ. 2015;116(1):1-22.
4. Hou K, Xue C, Zhang L. Digesting anomalies: An investment approach. Rev Financ Stud. 2015;28(3):650-705.
